# Synchrotron X-ray Studies of the Structural and Functional Hierarchies in Mineralised Human Dental Enamel: A State-of-the-Art Review

**DOI:** 10.3390/dj11040098

**Published:** 2023-04-07

**Authors:** Cyril Besnard, Ali Marie, Sisini Sasidharan, Robert A. Harper, Richard M. Shelton, Gabriel Landini, Alexander M. Korsunsky

**Affiliations:** 1 MBLEM, Department of Engineering Science, University of Oxford, Parks Road, Oxford OX1 3PJ, Oxfordshire, UK; 2 School of Dentistry, University of Birmingham, 5 Mill Pool Way, Edgbaston, Birmingham B5 7EG, West Midlands, UK

**Keywords:** synchrotron X-ray radiation, human tooth, enamel, caries, multi-modal analysis

## Abstract

Hard dental tissues possess a complex hierarchical structure that is particularly evident in enamel, the most mineralised substance in the human body. Its complex and interlinked organisation at the Ångstrom (crystal lattice), nano-, micro-, and macro-scales is the result of evolutionary optimisation for mechanical and functional performance: hardness and stiffness, fracture toughness, thermal, and chemical resistance. Understanding the physical–chemical–structural relationships at each scale requires the application of appropriately sensitive and resolving probes. Synchrotron X-ray techniques offer the possibility to progress significantly beyond the capabilities of conventional laboratory instruments, i.e., X-ray diffractometers, and electron and atomic force microscopes. The last few decades have witnessed the accumulation of results obtained from X-ray scattering (diffraction), spectroscopy (including polarisation analysis), and imaging (including ptychography and tomography). The current article presents a multi-disciplinary review of nearly 40 years of discoveries and advancements, primarily pertaining to the study of enamel and its demineralisation (caries), but also linked to the investigations of other mineralised tissues such as dentine, bone, etc. The modelling approaches informed by these observations are also overviewed. The strategic aim of the present review was to identify and evaluate prospective avenues for analysing dental tissues and developing treatments and prophylaxis for improved dental health.

## 1. Introduction

**Figure 1:**
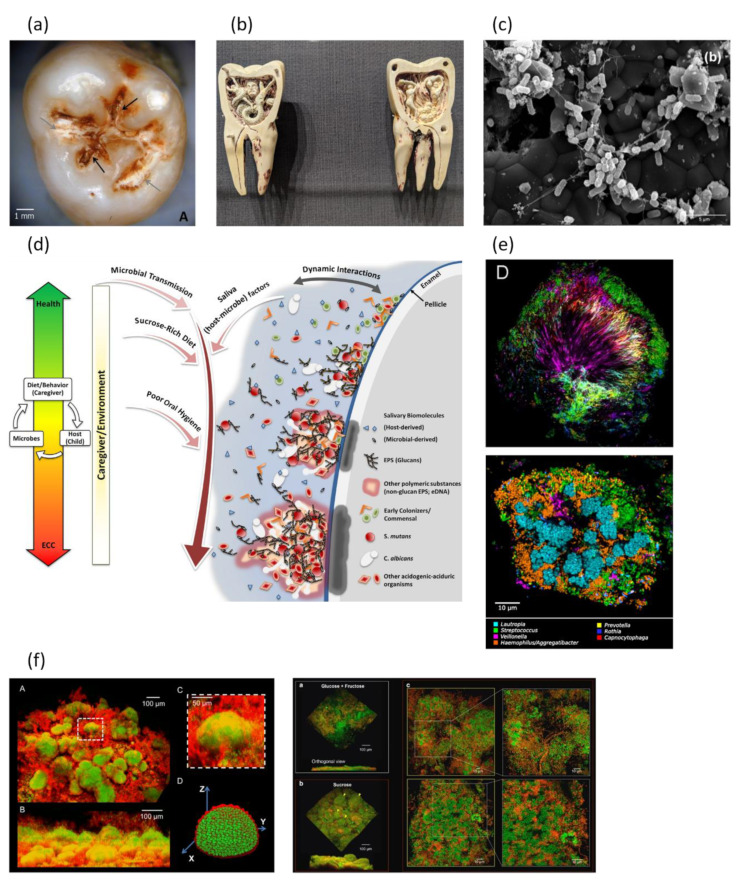
Imaging and illustration of dental caries, *Streptococcus mutans* and biofilms. (**a**) Photographic image of the surface of a tooth with dental caries showing the visual appearance of dental caries (adapted from [12] with permission. Copyright 2015, John Wiley and Sons). (**b**) Photographic image for the illustration of the dental caries image as a replica toothworm sculpture model of the 18th century original owned by the German Dental Association, image courtesy of the Dr. Samuel D. Harris National Museum of Dentistry, Baltimore, MD, USA. (**c**) SEM image of *Streptococcus mutans* (adapted from [13], license https://creativecommons.org/licenses/by/3.0/, accessed on 30 March 2023). (**d**) Schematic of the dynamic interactions occurring in early childhood caries (adapted from [14] with permission. Copyright 2016, John Wiley and Sons). (**e**) Confocal imaging of biofilms reused from [15] and (**f**) 3D imaging of *Streptococcus mutans* and EPS matrix, (adapted from Hwang G. et al. and Xiao J. et al. [16,17], license http://creativecommons.org/licenses/by/4.0/, accessed on 30 March 2023 and license http://creativecommons.org/licenses/by-nc-nd/4.0/, accessed on 30 March 2023 respectively). For details about the colour codes, see the publications.

Caries remains a debilitating condition that lacks adequate prevention and treatment and that demands further research to overcome its detrimental impact on global health. The disease had a global prevalence of around 2.3 billion in 2017 (in permanent teeth) [1]. In addition to the clinical effects, such as pain and discomfort, aesthetic issues (Figure 1a), and eventually tooth loss, it constitutes a huge economic burden, estimated to be billions of USD worldwide in necessary treatments [2,3]. Historically, caries (“decay” from Latin word *cariēs* [4]) was described as worms in the tooth [5,6,7], Figure 1b. The presence of worms was discussed by Fauchard in 1746 [8] “*mais je crois en m*ê*me tems, que ce ne font pas ces vers qui rongent & qui carient*” (p. 131), in his description of how this disease damages the tooth and affects the lives of people.

Understanding the mechanism of caries development requires tracing the pathways of the biological, chemical, and structural processes that unfold progressively from the microbial and crystal level up to the macroscopic scale [9]. This necessarily engenders the need to visualise and understand tissue organisation and function, along with its interaction with the microbial and chemical environment, through static and dynamic studies. Synchrotron-based studies offer unique tools for this purpose, due to the versatile interaction of X-ray photons with the organic and inorganic tissue components.

This review is organised as follows: The first section introduces the structure of the enamel (and dentine) to the degree necessary to understand the reported studies. This is followed by a description of dental caries disease and its causative factors, including the nature and organisation of biofilm and its effects on the enamel, followed by a discussion of existing strategies for remineralisation. The second section provides a brief overview of synchrotron facilities, followed by a description of the application of synchrotron methods to dental tissue studies: diffraction (scattering), imaging (including tomography and ptychography), and spectroscopy. Each sub-section is illustrated with examples from previous and recent developments and ongoing research. This review summarises the studies using synchrotron techniques for structural, imaging, and chemical analyses. The utility of these methods is emphasised in terms of bringing new insights, and the combined use of multiscale correlative techniques is highlighted. This comprehensive review will be of interest to a wide network of researchers and clinicians in the field of cariology, pharmaceutical industry, biomedical engineering, and nanodentistry [10,11].

## 2. Demineralisation and Remineralisation of Teeth

### 2.1. Tooth Structure

Human teeth have a complex anatomical structure [18,19,20,21,22], consisting of an outermost layer of a very hard acellular material [23] called enamel covering the crown of the tooth, i.e., the visible part of the tooth in the mouth. Enamel is supported by a less mineralised tissue called dentine. The junction between the two is known as either the amelo-dentinal junction (ADJ) or dentino-enamel junction (DEJ) [24,25]. The core of the tooth is a cellular tissue called the dental pulp [26], which is responsible for the delivery of nutrients [27] to odontoblasts and also contains nerves and blood vessels [18,26]. In the pulp, odontoblasts are the cells responsible for the formation and maintenance of the dentine [28]. The root anchors the tooth [29] to the jaw bones and is covered by another mineralised tissue (cementum) [30] that plays a crucial role in mechanical tooth retention and support [31]. Cementum is composed of two layers of cellular and acellular cementum [32]. The interface between cementum and dentine is known as the cemento-dentinal junction (CDJ) [33]. This complex hierarchical structure of the tooth provides a rich source of insights and ideas for the development of new materials via biomimetic research [34]. The present review mainly focuses on enamel, the most highly mineralised and hardest tissue in the human body.

#### 2.1.1. Enamel

**Figure 2:**
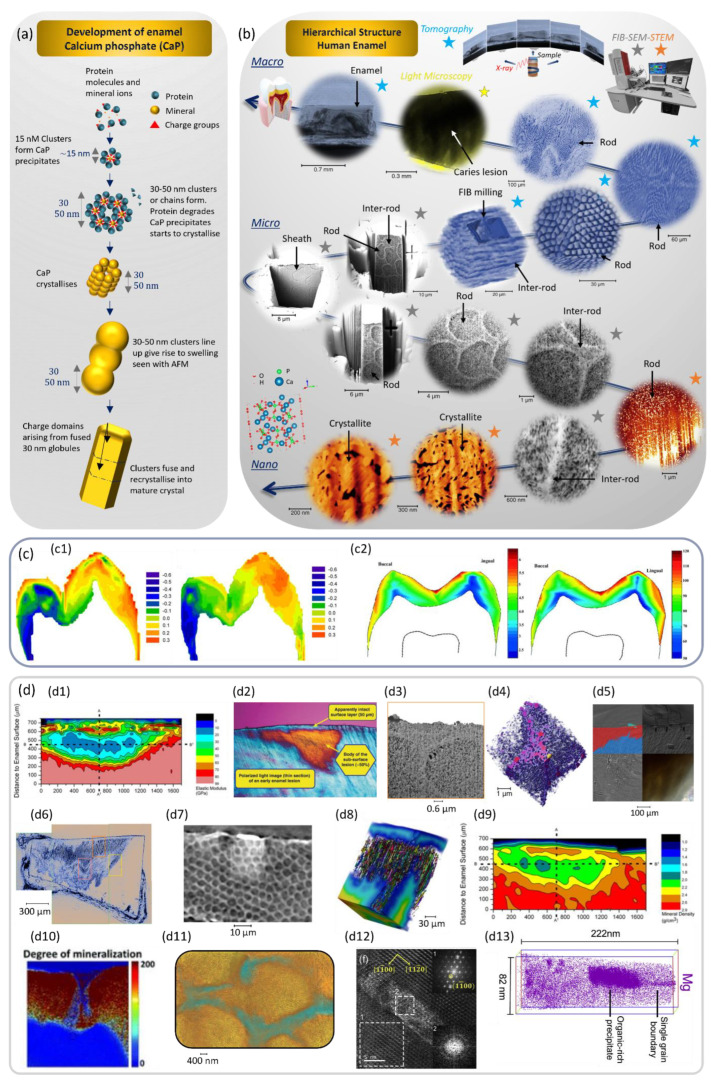
Hierarchical structure of human enamel and properties with and without caries. (**a**) Schematic of the mineralisation process of HAp from protein, mineral, and ions (reproduced from Robinson C. et al. [47], Copyright © 2017, license https://creativecommons.org/licenses/by/4.0/ accessed on 30 March 2023). (**b**) Synchrotron and electron microscopy images of the structure of enamel from macro to nano scale in a carious enamel sample (modified from [39], license https://creativecommons.org/licenses/by/4.0/ accessed on 30 March 2023). (**c**) Characterisation of non-carious enamel, (**c1**) lattice parameter a and c distribution in enamel (adapted from [89] with permission. Copyright 2007, Elsevier). (**c2**) Distribution of hardness and Young’s modulus in enamel (adapted from [133] with permission. Copyright 2002, Elsevier). (**d**) Characterisation results of carious enamel samples using various techniques of (**d1**) elastic modulus mapping from a white spot lesion (adapted from [134] with permission. Copyright 2010, Elsevier). (**d2**) Polarised light microscopy (adapted from [135] with permission. Copyright 2008, John Wiley and Sons). (**d3,d4**) SEM image and 3D rendering of slice and view of a FIB milled cross-sections of carious enamel, showing preferential demineralisation (adapted from [39], license https://creativecommons.org/licenses/by/4.0/ accessed on 30 March 2023). (**d5**) Correlative imaging with various imaging modes (light, electron microscopy and X-ray synchrotron) (adapted from [39], license https://creativecommons.org/licenses/by/4.0/ accessed on 30 March 2023). (**d6,d7**) Virtual slices from synchrotron tomography data showing a carious lesion in enamel (adapted from [39], license https://creativecommons.org/licenses/by/4.0/ accessed on 30 March 2023). (**d8**) 3D rendering of carious enamel and details of the spacing between rods (adapted from [57] with permission. Copyright 2021, Elsevier). (**d9**) Map of the mass density of a white spot lesion (adapted from [134] with permission. Copyright 2010, Elsevier). (**d10**) Raman spectroscopy mapping of enamel and dentine with a carious lesion (reproduced from [136] with permission from the Royal Society of Chemistry, license https://creativecommons.org/licenses/by/3.0/ accessed on 30 March 2023). (**d11**) Combined image of XRF showing calcium K_α_ fluorescence intensity and ptychography of human carious enamel, highlighting the correlation between structural and chemical analysis (adapted from [78], license https://creativecommons.org/licenses/by/4.0/ accessed on 30 March 2023). (**d12**) TEM image showing the dissolution of the core of the crystallite and amorphous central dark line (adapted from [88] with permission. Copyright 2020, Elsevier). (**d13**) APT of carious enamel with the 3D distribution of Mg (adapted from [88] with permission. Copyright 2020, Elsevier).

Enamel has a unique hierarchical structure [9,35,36,37,38,39,40,41]. It is mainly composed of inorganic mineral (85% by volume), hydroxyapatite (HAp), a small percentage of proteins, and water (12% by volume) [42]. The organic content remaining from the formation stages of the enamel plays an important role in maintaining the enamel structure and continuity [43,44,45,46]. Enamel is mainly composed of HAp crystallites deposited by ameloblasts during development stages, which have been suggested to follow the arrangement of protein and mineral clusters [47] (Figure 2a) and nanoribbons [48,49,50,51]. Multiple HAp crystallites are bundled together and arranged in ‘rods’ [52] of about 5 μm diameter with a cross-section shaped as a ‘keyhole’. These rods are separated by an inter-rod substance of around 2 μm thickness [39,53,54,55,56,57,58,59,60,61], Figure 2b. The bulk of the enamel is prismatic in nature; however, a prism-less region (referred to as ‘aprismatic enamel’) at the surface of the tooth can sometimes be found [62,63,64,65]. A very thin region between rods, referred to as the sheath, has also been mentioned in the literature [66,67,68,69,70]. The nanocrystallites of HAp forming the rods are arranged in a hexagonal structure [71] of ~26.3–36 nm width and ~68.3 nm thickness on average and more than 100 μm in length [72,73,74]. With the use of transmission electron backscatter diffraction, some HAp crystallites have been seen to be arranged in chains with a similar orientation [75,76]. Inhomogeneity in the orientation of the crystallites in rods and inter-rods was also reported and visualised using transmission electron microscopy (TEM), polarization-dependent imaging contrast (PIC) map analysis, and ptychography [55,77,78]. The HAp composition in enamel is different from that of pure HAp (Ca_10_(PO_4_)_6_(OH)_2_) and can contain additional trace elements, e.g., Se and F [71,79,80,81,82,83,84,85,86,87,88] (additional details in SI—Table S1, and there are references in the SI). Besides its complexity of composition, the crystal lattice is also inhomogeneous. The crystal lattice parameters ‘a’ and ‘c’ of HAp in enamel are dependent on various parameters, such as the location in the teeth, age, and the presence of trace elements [81,82,89,90,91], Figure 2c.

Recently, nanoscale technique atom probe tomography (APT) studies have provided new insights into the structure of human enamel, with early studies on rodent teeth followed by human teeth, providing 3D elemental analysis at nano-resolution levels. In rodents, an ionic distribution with a high content of magnesium (Mg) in the grain boundary of the enamel structure, as well as Mg in the apatite crystal lattice, was revealed [92]. The enrichment of elements observed in the grain boundaries suggested this plays a role in the process of tooth decay. The solubility of enamel was suggested by LeGeros R.Z. et al. and Gordon L.M. et al. to be influenced by the composition of impurities, such as Mg^2+^ and F^−^ [81,92]. The use of APT provided the 3D distribution of elements in enamel at the nanoscale and enabled analysis of nano-channels, which could influence the solubility of the enamel during acid dissolution. Mg-amorphous calcium phosphate (ACP) was proposed as an intergranular phase in normal rodent enamel; and in another experiment, by comparison with an iron-rich enamel named pigmented enamel, it was hypothesised that the presence of Mg-ACP lowers the acid resistance of enamel in comparison with iron phosphate. Human enamel was also characterised using APT, revealing its atomic level composition, and at the interface regions between HAp crystallites, Mg-rich ACP was found [93,94]. 

Other structural features of enamel include the striae of Retzius and Hunter–Schreger bands. Retzius striae arise from temporal variations in the secretory activity of ameloblasts during enamel formation [23,95,96,97], while Hunter–Schreger bands [98,99] arise as an optical effect on sections of enamel, due to changes in the direction of the enamel rods across the tissue thickness from the surface to the DEJ, which results in a weaving into other rods, which is thought to improve the mechanical properties of enamel [100,101]. The Hunter–Schreger bands can be visualised using optical microscopy and scanning electron microscopy (SEM) [102,103,104,105,106] (they are also present in animal enamel, e.g., bear [107], dog [108], and rhinoceros [109]), where the wavy pattern can be visualised using tomography [39,57,109]. The distribution of the striae of Retzius and their width are not homogeneous, e.g., their spacing increases with enamel depth [62,95]. In a study of teeth from apes and modern humans, a spacing ranging from 25 to >150 μm was reported [110]. The rods in the region of the striae of Retzius appeared enlarged in comparison with other locations [111]. In the rods, there have been observations of cross striation spacing [111] along the c-axis of the rods of a few microns, linked to the rhythmic arrangement of the ameloblast. A periodicity of cross striation with a mean of 5.3 μm was observed using SEM [62,112,113] and 2.7 µm using Raman spectroscopy [114]. The complex hierarchical structure of enamel is summarised in Figure 2b, with SEM and synchrotron images of human enamel, showing some of the structural details.

#### 2.1.2. Dentine

Dentine is present beneath the layer of enamel in the crown and cementum in the roots and is separated by the dentino-enamel and the dentinocemental junctions. Dentine is less mineralised compared with enamel and contains around 47% by volume of mineral phase, 33% by volume of lipid and protein, and 20% by volume of water [42]. It also possesses a complex structure, with tubules containing cellular extensions of odontoblasts in the pulp, as well as unmyelinated nerve fibres [115]). The tubules are lined by peritubular dentine (PTD containing HAp and an organic phase), while a collagen matrix of intertubular dentine (ITD) is present between the tubules [116,117,118,119]. The density and diameter of tubules are not homogeneous across the dentine thickness [120,121]. From the superficial to deep dentine, the diameter of the tubules increases from around 2 to 4 μm [122], or 2 to 3 μm in another study [123]. Another study using atomic force microscopy (AFM) revealed that the crystallites were larger in ITD than in PTD [124]. 

The DEJ, present between dentine and enamel, has important properties to compose the interface between the two different tissues [125,126]. The DEJ has been characterised using various analytical techniques, including nanoindentation, AFM, digital image correlation, TEM, and dynamic mechanical analysis, providing details on its dimensions and mechanical properties [125,126,127,128,129,130,131,132].

### 2.2. The Role and Structure of Biofilm in Caries Disease

The complex structure of dental enamel contributes to its outstanding properties, e.g., resistance to hot and cold thermal shocks, and mechanical properties able to withstand intense mastication forces, as well as self-healing properties, as recently detailed [137]. Enamel is a hard tissue with a hardness value of ~4 GPa, which varies as a function of the location in the tooth at the macro scale, as well as between rods, inter-rods, and sheath [67,131,133,138,139,140,141], Figure 2c. The enamel hardness and Young’s modulus are found to be decreased in carious lesions [134,142], Figure 2d. These unique properties are due to the specific crystal lattice, arrangement of atoms, and chemical composition, which require advanced techniques for characterisation. Despite these outstanding properties, dental tissues are prone to demineralisation by acids, resulting in erosion and/or caries in enamel, dentine, and cementum [117,143,144,145,146,147,148,149,150]. For the purpose of this review, it is important to differentiate dental caries from enamel erosion. Dental caries is a chronic multifactorial process resulting in demineralisation (of enamel, dentine, or cement) by acids from bacteria in adherent biofilms or plaque, whereas dental erosion is the irreversible loss of enamel structure due to the effect of acids either from the diet (intrinsic) or acid reflux (extrinsic) [151,152,153]. In addition, in dentine and cementum caries, the more prominent organic matrix is broken down by proteolytic enzymes produced by bacteria. 

A major player in the aetiology of dental caries is *Streptococcus mutans,* one of several bacterial species that can ferment dietary sucrose [13,151,154] (Figure 1c), to develop complex biofilms and resulting in acid production [155,156,157,158]. Biofilms have a complex architecture and physical properties, with complex bacterial aggregates suspended in a protecting layer of exopolysaccharides (EPS), Figure 1d–f.

The acid produced by bacterial biofilms results in a low environmental pH. Understanding the profile of pH in biofilms and developing treatment strategies aimed at altering the biofilm or decreasing the effects of the acid on the dental tissues seem obvious approaches for investigation. An in situ analysis of the pH in biofilms produced by *Streptococcus mutans* revealed that they withstand neutralisation and can maintain an acidic environment, referred to as an ‘acidic core’. A 3D view of biofilms with microcolonies of bacteria embedded in the EPS-matrix is shown in Figure 1f. The localised acidic core in the microcolony 3D structure can lead to the formation of caries, even after acid neutralisation of the colonies [16]. These results were also supported by a recent study where enamel lesions were correlated with the location of biofilm, in which the pattern of pH variation in the biofilm was determined as a function of time during neutralisation with a solution of pH 7 and re-acidification with glucose [17]. In parallel to the study of pH, the molecular composition of biofilms has been also investigated using synchrotron radiation on samples with different carious conditions and treated with prophylactic agents, focusing on the dynamic process occurring between the oral fluid and biofilm [159]. In one study, the importance of the EPS matrix in the presence of an acidic core was demonstrated. The EPS matrix was treated with enzymes that degrade the EPS while observing the pH profile, to evaluate their effect on the reduction of the biofilm. The study showed significant neutralisation of the pH in the interior of the 3D microcolonies in the treated samples compared with a control [16]. More promising findings on the alteration of the EPS matrix in biofilms were reported in another study; following the application of shear stress to the biofilms using a mechanical strength tester, the adhesion of the biofilm on a holder was reduced in the biofilm treated with enzyme dextranase in comparison to the normal condition [155]. The use of an assembly of nanoparticles under magnetic fields has been shown to be promising for the removal of biofilms [160]. The use of mangostin (αMG), a xanthone purified from a plant, as an antimicrobial agent against planktonic *Streptococcus mutans* was also reported to be effective in the reduction of the EPS matrix [161]; however, its mode of action is not fully understood. Rather than targeting the EPS matrix, other strategies have also studied a direct attack on the bacteria. Thus, studies on dental resin components, dental monomers, and initiators decreasing the activity of the bacteria [162], as well as the use of nanoparticles in vitro and in vivo in rats to limit the growth of the *Streptococcus mutans,* have provided useful insights [163]. Additional details on antimicrobial agents can be found in [164]. In addition to acid, it was shown that some genes could influence the enhancement of the cariogenic effect on enamel [165,166]. 

### 2.3. Effect of Dental Caries on Enamel Structure

The dissolution of the mineral structure of enamel by acid leads to the dissolution of specific locations in rods and HAp crystallites [9,39,57,142,167,168,169]. Various investigations have been carried out on natural and artificial caries using advanced techniques, to provide a better understanding of the demineralisation process [170,171,172,173]; for instance, the preferential dissolution along the Hunter–Schreger bands [145], striae of Retzius [174,175,176,177,178], and sheath [179]. A detailed description of enamel structure in 2D and 3D using several imaging techniques has recently been reported for human carious enamel, showing the complex demineralised structures [39]. Different regions have been traditionally reported, using optical microscopy and radiography, and referred to as ‘body of the lesion’, ‘surface’, ‘translucent’, and ‘dark’ zones of enamel caries [135,174,180,181,182] (Figure 2d); however, the presence of additional zones should not to be omitted [39,183]. The surface zone is a critical region of enamel (in direct contact with the environment and dental plaque), which has not yet been totally understood and characterised. In the carious region, preferential demineralisation locations have been observed in rods, inter-rod substances, and crystallites [57,184,185,186,187]. Correlative imaging using laboratory and synchrotron analysis recently provided new insights into the micro and nano histology of human carious lesions, such as localised porosity analysis in 2D and 3D (using synchrotron tomography and focused ion beam (FIB)-SEM characterisation), in the lesion, surface, and in non-carious regions, revealing the internal details of the carious structure, which were not observed with optical microscopy [39,57]. This was also correlated with electron microscopy analysis and the details of porosity in various regions of enamel from FIB milled cross-sections, as well as FIB ‘slice and view’ process. The porosity in the rods was visualised with different imaging modalities, Figure 2d. Significant differences in porosity were found in the body of the lesion compared to the other zones. Using tomography, measurement of the porosity (based on grey values) of the demineralised and non-demineralised regions was carried out [57], as well as their density variation and the decrease in the carious lesion [142], Figure 2d. In addition, the chemical composition was also investigated in carious and normal enamel using Raman spectroscopy/Raman peaks [136,188], as well as the difference found in calcium K_α_ fluorescence intensity in rod and inter-rod in carious enamel using X-ray fluorescence spectroscopy (XRF), Figure 2d. The underlying cause of enamel susceptibility to acids has sparked interest in investigating the possible points of weakness that initiate demineralisation. For this purpose, researchers have investigated nano-characterisation techniques (e.g., TEM, APT) and synchrotron X-ray absorption spectroscopy (XAS), to provide details about potential locations of the initiation of demineralisation, Figure 2d. Such locations include a high angle grain boundary (113.5°) between HAp crystallites, a structural defect located in the crystallite HAp referred to as ‘central dark line’ (CDL) with a width of ~1 nm [189] (the CDL has been extensively investigated [190,191,192]) (Figure 2d), organic phase [88] (Figure 2d), chemical composition creating residual stress [193] and defects [9,194], or the striae of Retzius previously mentioned. 

Surface enamel can undergo remineralisation from Ca and P ions in saliva (resulting in it being less demineralised than the body of the lesion) [134,146]. This phenomenon occurs through the physico-chemical processes of diffusion, dissolution, and precipitation [195]. Previous biomineralisation research focused on ex situ studies [196,197]; however, in situ enamel remineralisation studies that lead to complete regeneration of enamel remain challenging.

### 2.4. Dental Remineralisation Strategies

The traditional dental caries treatment is carried out by removing diseased tissue and filling it with restorative materials, but it would be desirable to replace this with repair strategies based on biomimetic remineralisation (inspired by nature [198]). Such a task would require the aim being rebuilding enamel with a similar texture and high resistance to acid, and offering remineralisation pathways from the surface to the depth of cavities. For additional details, readers are referred to specific reviews on remineralisation [197,199,200,201,202,203,204,205,206,207,208,209,210,211,212,213,214,215] and studies of the various types of materials used for enamel repair, including ceramics [216], polymers [217], bioinspired composites [218], bioinspired nanocrystals [219], bioglass [202], and coating with calcium phosphate ion clusters [220].

In addition to the ionic contents of saliva as an internal source of remineralising elements [221,222], fluoride has been known to bind to HAp to form fluoro-hydroxyapatite or fluoro-apatite (FAp), with a higher resistance to acid dissolution than HAp [223,224]. On the other hand, there has been a focus on strategies using synthetic peptides (chains of amino acid) [225] to induce the formation of hydroxyapatite [226], as well as proteins [226,227,228,229,230] involved in the formation of dental enamel during amelogenesis [23]. A study on synthetic recombinant amelogenins using TEM and diffraction showed that samples with a C-terminal domain were able to facilitate the formation of crystallites with improved orientation, rather than being randomly orientated [231]. Amelogenin constitutes a major part of the proteins in the enamel matrix that remain after maturation, and it plays a role in the organisation of the hydroxyapatite during the enamel formation [23,46,226,232,233]. A study on amelogenin-deficient null mouse showed no effect on the inhibition of the growth of hydroxyapatite, but a lack of prismatic structure of the enamel was observed in comparison to a control sample (wild type) [234]. Similarly, in amelogenin knock-out mice, the enamel crystallites were comparable to those in the previous study, although the organisation and the crystallite size were altered, the aspect ratio (width to thickness of the crystallite) was found to be conserved. That study also showed that using recovery of the amelogenin protein resulted in HAp crystallites of normal size [232]. Researchers have investigated the proteins that play a role in the growth and structure of the hydroxyapatite, in order to design and build customised peptides that include a key sequence, such as the terminus part of the amelogenin rP172 [228] (full-length recombinant), P26 and P32 (32 amino acid residues). Enamel added to a peptide and an artificial remineralisation solution induced the formation of a new layer of aprismatic enamel. While the crystal orientation was analysed with diffraction and the structure visualised with microscopy, its resistance to acid was not reported. The shADP5 peptide (amelogenin-derived peptides, and “h” stands for “human” and “s” for “short”) based from rM180 (180 amino acid-long human amelogenin protein) was also studied. This showed a significant improvement in the remineralisation with the production of a layer observed with SEM without significant porosity and a higher microhardness (nanoindentation measurements) compared with the samples treated with a control solution (although still lower than normal enamel) [229]. These studies, however, did not investigate acid dissolution. In a recent study, the use of intrinsically disordered protein elastin-like recombiners and cross linking was also investigated, and remineralisation was achieved with a similar needle-like structure to that in enamel [227]. Using a different approach, calcium-phosphate compounds integrated with peptides have also been tested in remineralisation studies. Amorphous calcium phosphate (ACP) [230] was synthesised and functionalised with elastin-like polypeptide E125 acid groups, to aid in the remineralisation process. The texture, of the new layer, linked to the degree of orientation of the crystal of hydroxyapatite was reported to be closer to normal enamel, in comparison to the control solution that used L-glutamic acid with a different orientation characterised with diffraction. The structure of the remineralised layer was visualised with SEM, with the observation of crystal growth. The formation of artificial enamel was also reported to be possible using phase transformation of ACP, leading to the formation of HAp crystallites [235]. ACP was also used with casein phosphopeptide-ACP with stannous fluoride [236], which resulted in an improvement in the remineralisation of the enamel, as seen with transverse microradiography. It has been reported that HAp@ACP nanoparticles in the presence of amino acid glycine aided in the guided growth of HAp crystals [237]. The combination of synthetic ACP nanoparticles and peptides was also investigated, a new layer was found on the enamel using SEM, and the texture determined with X-ray diffraction was found to be close to the control enamel [238]. Other studies reported the use of chitosan and the enamel matrix derivative protein amelogenin and peptides, which lead to the formation of new layers of material [239,240]. 

The general aims of all these strategies are to maintain, repair, or improve the original properties of normal enamel and dentine and improve the acid resistance of dental hard tissues. To summarise, remineralisation studies require a comprehensive understanding of normal enamel, in terms of its formation through amelogenesis, its mechanical properties, structure, and chemistry, at each scale before, during, and after demineralisation, to aid in the understanding of the process of biomineralisation. On the other hand, any proposed remineralisation solution/material and newly deposited structures should also be characterised using a comprehensive analysis of the properties of the newly mineralised material, to evaluate whether the repair mimics natural enamel or has any enhanced properties.

## 3. Synchrotron (Circular Particle Accelerator)

Synchrotron X-ray radiation is characterised by its coherence and high brilliance (several orders of magnitude higher in comparison with the sun [241]), which can be exploited to enable the visualisation of complex structures. The discovery of X-rays has a long history [242,243], starting with the breakthrough in Röntgen’s work [244,245,246]. The principle of synchrotron radiation is based on the emission of X-rays from the change of direction of accelerated electrons. These X-rays are then directed and used to analyse the material of interest through several techniques, either individually or in a correlative processing mode, for multidisciplinary applications [247]. The first synchrotron radiation was detected in 1947 [248,249,250]; but currently, about 50 synchrotron facilities are operating in the world, contributing to an outstanding amount of research work [251] along with the continuous improvement of analytical approaches [252]. The modern synchrotron offers the versatility of utilizing customised experimental setups, which can be categorised based on the type of detector and relevant setup; the energy in use, either soft or hard X-rays [253,254] (in vacuum or air or liquid); the presence of magnetic fields or temperature control; and the type of monitoring process (static or dynamic analysis) and equipment [255]. These facilities have been used to study dental tissue and will be covered in this review.

In this regard, in this second part of the review, the main focus is to give an overview of the use of synchrotron X-ray applications in human dental research (for animal species, numerous studies have been carried out on a variety of species and fossils [256,257,258,259,260,261,262,263,264,265,266,267,268,269,270,271,272,273,274,275,276,277,278,279], as well as studies on biofilms [280,281,282,283]). This review was performed by searching several platforms, including ScienceDirect, Web of Science, and SciFinder. Major databases were checked using the keywords synchrotron, tooth, caries, carious, enamel, and remineralisation (non-exhaustive list). The results of the search were compiled into databases and used to generate visualisation networks with the software VOSviewer v. 1.6.19 [284]. The details of the synchrotron applications and techniques in this review complement those of Hwu Y. et al. [285] and are an important addition to previous reviews on enamel [56,57,286], as well as synchrotron studies for engineering applications [247], X-ray nano (micro) probes [287], techniques [288], materials [289], soft matter [290], bone [291], biological materials [292], nano–bio interface [293], biominerals [294], biological samples [295], radiation of biological samples [296], cells [297], biomedicine [298], trace elements [299], heavy metals [300], chemical imaging [301], cathodes [302], and palaeontology [267].

**Figure 3:**
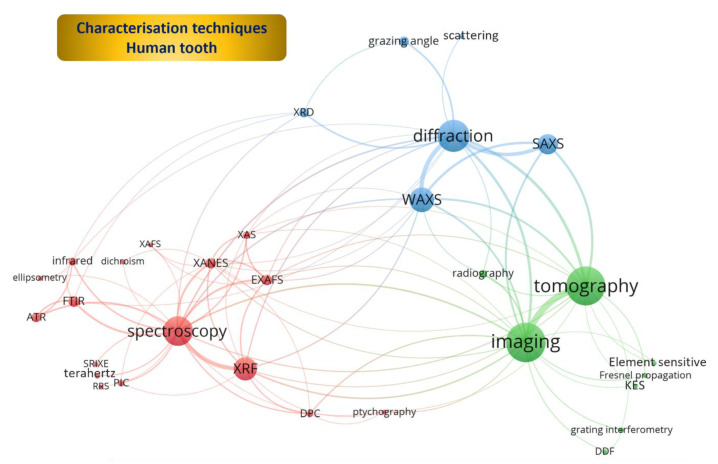
Visualising networks of synchrotron-based techniques used for research on human dental tissues (see also in Figure 4). A bibliometric networks map was created from the cited studies from a period of 39 years (1984–2023). The map was created based on text data and term co-occurrence with the software VOSviewer version 1.6.19 [284]. The data were extracted from Endnote software in ‘.txt’ format and manually edited to arrange the studies by techniques. In total, 31 terms were considered based on these studies [11,31,39,56,57,77,78,89,90,121,169,170,171,172,173,175,193,195,247,266,286,294,299,303,304,305,306,307,308,309,310,311,312,313,314,315,316,317,318,319,320,321,322,323,324,325,326,327,328,329,330,331,332,333,334,335,336,337,338,339,340,341,342,343,344,345,346,347,348,349,350,351,352,353,354,355,356,357,358,359,360,361,362,363,364,365,366,367,368,369,370,371,372,373,374,375,376,377,378,379,380,381,382,383,384,385,386,387,388,389,390,391,392,393,394,395,396,397,398,399,400,401,402,403,404,405,406,407,408,409,410,411,412,413,414,415,416,417,418,419,420,421,422,423,424,425,426,427,428,429,430,431,432,433,434,435,436,437,438,439,440,441,442,443,444,445,446,447,448,449,450,451,452,453,454,455,456,457,458,459,460,461,462,463,464,465,466,467,468,469,470,471,472,473,474,475,476,477,478,479,480,481,482,483,484,485,486,487,488,489,490,491,492,493,494,495,496,497,498,499,500,501,502,503,504,505,506,507,508,509,510,511,512,513,514,515,516,517,518,519,520,521,522,523,524,525,526,527,528,529,530,531,532,533,534,535,536,537,538,539,540,541,542,543,544,545,546,547,548,549,550,551,552,553,554,555,556,557,558,559,560,561,562,563,564,565]. This combined most of the studies we could find (to the best of our knowledge) on human teeth analysed with synchrotron radiation, along with a few on archaeological samples, e.g., Hominin and Homo juvenile. The acronyms are detailed in the manuscript.

**Figure 4:**
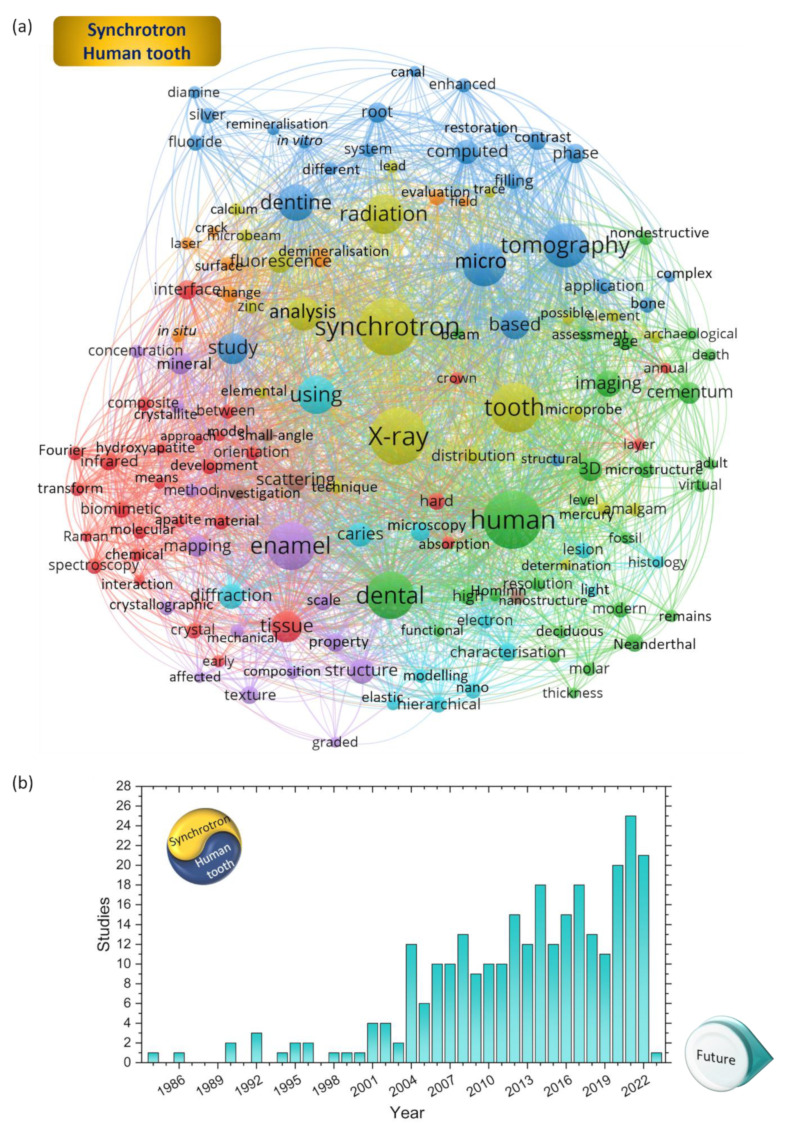
Visualising bibliometric networks of the studies on human teeth using synchrotrons and yearly distribution of the studies. (**a**) Bibliometric network map from studies (human teeth, and in addition, a few studies on archaeological samples e.g., Hominin, and Homo juvenile) from a period of 39 years (1984–2023), from the first report on microradiography in 1984 to a study of 2023. The map was created based on text data and term co-occurrence with the software VOSviewer version 1.6.19 [284]. The data were extracted from Endnote software (Clarivate) in ‘.txt’ format and manually edited to match the reading format and correlation of words; for example, British spelling was applied when translating non-English titles and acronyms were generally written in full length. The minimum number of occurrences of a term was set to four, and in total 140 terms were used based on these studies [11,31,39,56,57,77,78,89,90,121,169,170,171,172,173,175,193,195,247,266,286,294,299,303,304,305,306,307,308,309,310,311,312,313,314,315,316,317,318,319,320,321,322,323,324,325,326,327,328,329,330,331,332,333,334,335,336,337,338,339,340,341,342,343,344,345,346,347,348,349,350,351,352,353,354,355,356,357,358,359,360,361,362,363,364,365,366,367,368,369,370,371,372,373,374,375,376,377,378,379,380,381,382,383,384,385,386,387,388,389,390,391,392,393,394,395,396,397,398,399,400,401,402,403,404,405,406,407,408,409,410,411,412,413,414,415,416,417,418,419,420,421,422,423,424,425,426,427,428,429,430,431,432,433,434,435,436,437,438,439,440,441,442,443,444,445,446,447,448,449,450,451,452,453,454,455,456,457,458,459,460,461,462,463,464,465,466,467,468,469,470,471,472,473,474,475,476,477,478,479,480,481,482,483,484,485,486,487,488,489,490,491,492,493,494,495,496,497,498,499,500,501,502,503,504,505,506,507,508,509,510,511,512,513,514,515,516,517,518,519,520,521,522,523,524,525,526,527,528,529,530,531,532,533,534,535,536,537,538,539,540,541,542,543,544,545,546,547,548,549,550,551,552,553,554,555,556,557,558,559,560,561,562,563,564,565]. (**b**) Timeline bar chart of the distribution of studies [11,31,39,56,57,77,78,89,90,121,169,170,171,172,173,175,193,195,247,266,286,294,299,303,304,305,306,307,308,309,310,311,312,313,314,315,316,317,318,319,320,321,322,323,324,325,326,327,328,329,330,331,332,333,334,335,336,337,338,339,340,341,342,343,344,345,346,347,348,349,350,351,352,353,354,355,356,357,358,359,360,361,362,363,364,365,366,367,368,369,370,371,372,373,374,375,376,377,378,379,380,381,382,383,384,385,386,387,388,389,390,391,392,393,394,395,396,397,398,399,400,401,402,403,404,405,406,407,408,409,410,411,412,413,414,415,416,417,418,419,420,421,422,423,424,425,426,427,428,429,430,431,432,433,434,435,436,437,438,439,440,441,442,443,444,445,446,447,448,449,450,451,452,453,454,455,456,457,458,459,460,461,462,463,464,465,466,467,468,469,470,471,472,473,474,475,476,477,478,479,480,481,482,483,484,485,486,487,488,489,490,491,492,493,494,495,496,497,498,499,500,501,502,503,504,505,506,507,508,509,510,511,512,513,514,515,516,517,518,519,520,521,522,523,524,525,526,527,528,529,530,531,532,533,534,535,536,537,538,539,540,541,542,543,544,545,546,547,548,549,550,551,552,553,554,555,556,557,558,559,560,561,562,563,564,565] over the researched period.

**Figure 5:**
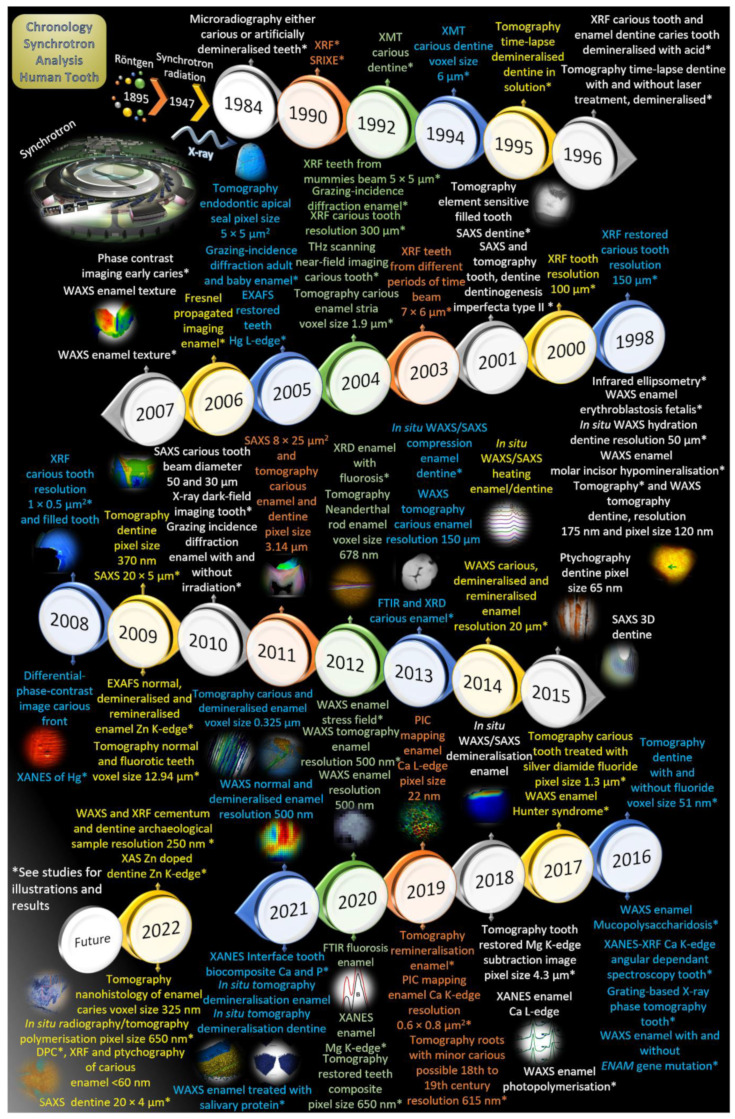
Timeline of the synchrotron studies carried out on human teeth. Timeline showing studies that have been performed on human teeth, with the details of some research done over the past decades covering different modalities and applications, e.g., WAXS, SAXS, tomography, X-ray fluorescence spectroscopy, and PIC mapping, the details in the figure are from these references [31,39,56,57,77,78,89,121,169,170,171,172,175,193,195,303,304,310,318,327,328,333,338,344,348,349,353,354,361,363,368,372,376,380,385,392,399,403,404,407,412,413,414,416,419,420,423,425,442,448,451,461,472,474,480,481,484,485,499,506,510,513,524,526,527,530,532,537,538,539,545,547,548,549,551,552,553,555,556,560,561]. Furthermore, illustrations of some results are shown in chronological order, images adapted from [545] with permission. Copyright 2001, Elsevier, adapted from [526] with permission. Copyright 2005, Elsevier, adapted from [89] with permission. Copyright 2007, Elsevier, reproduced from [499] with permission of the International Union of Crystallography, adapted from [480] with permission. Copyright 2010, Elsevier, adapted from [472] with permission. Copyright 2011, Elsevier, adapted from [451] with permission. Copyright 2012, Elsevier, adapted from [448] with permission. Copyright 2013, Elsevier, reproduced with permission from Sui T. et al. [425], adapted from [413], license https://creativecommons.org/licenses/by/4.0/ accessed on 30 March 2023, adapted from [416] with permission. Copyright 2015, Springer Nature, adapted with permission from [420]. Copyright 2015 American Chemical Society, adapted from [170] with permission. Copyright 2018, Elsevier, reprinted (adapted) with permission from [372]. Copyright 2018 American Chemical Society, adapted from [77], license http://creativecommons.org/licenses/by/4.0/ accessed on 30 March 2023, adapted from [348], license http://creativecommons.org/licenses/by/4.0/ accessed on 30 March 2023, adapted from [354] with permission. Copyright 2020, Cambridge University Press, adapted from [57] with permission. Copyright 2021, Elsevier, adapted from [333] with permission. Copyright 2021, Elsevier, adapted from [327], license https://creativecommons.org/licenses/by/4.0/ accessed on 30 March 2023, adapted from [304], adapted from [56], license https://creativecommons.org/licenses/by/4.0/ accessed on 30 March 2023, adapted from [39], license https://creativecommons.org/licenses/by/4.0/ accessed on 30 March 2023, adapted from [78], license https://creativecommons.org/licenses/by/4.0/ accessed on 30 March 2023. ‘*’ referred to as the studies where no illustration is added to the Figure. The image of the synchrotron used in the Figure, https://www.diamond.ac.uk/PressOffice/MediaResources.html accessed on 30 March 2023, ©Diamond Light Source, 2022.

This review covers different characterisation techniques, including diffraction (scattering), imaging, and spectroscopy, which will be described in the next sections. These modalities and their complementary associations in different applications are illustrated in Figure 3 using visualisation networks. These analytical methods have been used to study the structure, mechanical properties, and chemical properties of dental tissues (summarised in Figure 3 and Figure 4). This highlights that several fields of research and biological tissues have been investigated, covering a multitude of aspects of the tooth structure, its elemental analysis, and imaging techniques, with individual or combined techniques. In addition, the review shows potential connections between different fields of research. A timeline of synchrotron studies on human teeth and its various applications reported in the literature is presented in Figure 5, in chronological order, and shows that there have been ~286 research studies. The earliest, on carious or demineralised teeth, was conducted in 1984 [303], and since then there has been a rapid increase in the number of studies and complexity of experimental setups, including correlative analyses, nano analysis, and in situ experiments [77,304] (Figure 5).

### 3.1. X-ray Scattering—Diffraction

Wide-angle-X-ray scattering (WAXS) diffraction based on Bragg’s law [566] (Equation (1)) has been extensively used for the analysis of human dental tissues, including enamel with and without caries, affected with other diseases, and with and without artificial demineralisation–remineralisation [56,172,414]. This technique is especially suitable for studies on enamel, because of the mineral composition of tissue with HAp crystallites, from which the crystal planes of the sample diffract.

This technique is based on the formula:

(1) $$n\lambda =2d_{hkl}\sin\theta$$

were d indicates the interplanar spacing, hkl are the Miller indices, θ is half of the scattering angle, and n is the integer of the wavelength λ.

**Figure 6:**
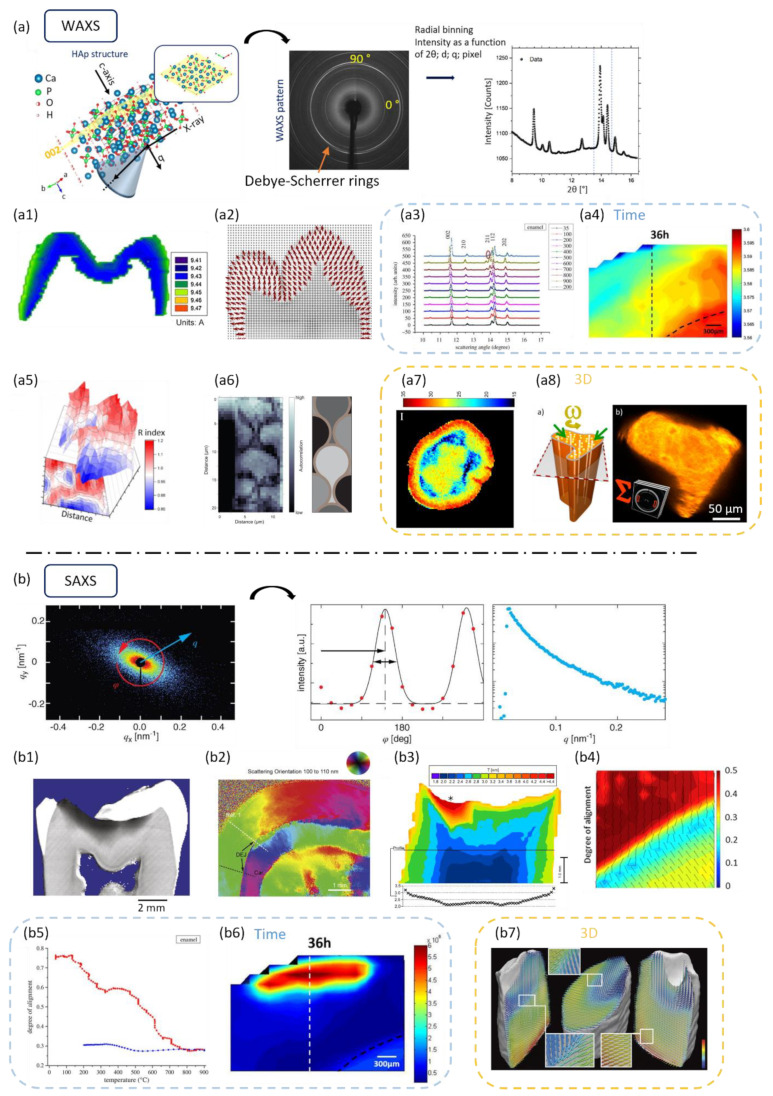
Illustration of WAXS and SAXS synchrotron application to human teeth. (**a**) Crystal structure of HAp and WAXS analysis with details of the signal and formation of the Debye–Scherrer ring, adapted from [56], license https://creativecommons.org/licenses/by/4.0/ accessed on 30 March 2023, and applications with (**a1**) distribution of the a-lattice parameter in dental human enamel (adapted from [90] with permission. Copyright 2013, Elsevier). (**a2**) The texture map of the hydroxyapatite crystallites in human enamel based on the (002) peak (adapted from [89] with permission. Copyright 2007, Elsevier). (**a3**) Diffractograms of enamel under heating and cooling treatments (reproduced with permission from Sui T. et al. [425]). (**a4**) Evolution of the d-spacing of human enamel during acid exposure (adapted from [170] with permission. Copyright 2018, Elsevier). (**a5**,**a6**) Sub-micron resolution maps of human enamel with and without demineralisation (adapted from [56], license https://creativecommons.org/licenses/by/4.0/ accessed on 30 March 2023, and adapted from [354] with permission. Copyright 2020, Cambridge University Press). (**a7**) Virtual slice from 3D diffraction tomography of human carious tooth showing nanocrystalline size in nanometres (adapted from [448] with permission. Copyright 2013, Elsevier). (**a8**) 3D diffraction tomography, showing the apatite orientations in human dentine with colour map assigned to the intensity (0 to 1 arb. units) adapted with permission from [420]. Copyright 2015 American Chemical Society). (**b**) SAXS analysis with the details of one SAXS pattern and interpretation of the pattern (adapted from [395] with permission. Copyright 2016, John Wiley and Sons). (**b1**) Map of SAXS transmission data from a carious tooth (adapted from [472] with permission. Copyright 2011, Elsevier). (**b2**) Map of the scattering orientation from a carious tooth (adapted from [173] with permission. Copyright 2014, Elsevier). (**b3**) Map of the distribution of the particle thickness in a carious tooth (adapted from [480] with permission. Copyright 2010, Elsevier). (**b4**) Map of the degree of alignment in dentine and enamel of HAp crystallites from a sample heated at 500 °C (adapted from [426], license https://creativecommons.org/licenses/by/3.0/ accessed on 30 March 2023). (**b5**) Distribution of the degree of alignment of the mineral in human enamel under heating and cooling treatments (reproduced with permission from Sui T. et al. [425]). (**b6**) Total intensity plot of SAXS data during acid demineralisation of a human tooth (adapted from [170] with permission. Copyright 2018, Elsevier). (**b7**) SAXS 3D of human dentine with the analysis of the collagen fibre orientation and average scattered intensity (colour bar 0.88–0.94 Å^−1^) with a resolution of ~100 µm (adapted from [416] with permission. Copyright 2015, Springer Nature).

The data generated from this technique, in particular, the 2D Debye–Scherrer rings with different integrations of the WAXS pattern, i.e., radial and/or azimuthal (Figure 6a) provide details on parameters including the phase, lattice parameters (Figure 6a), texture (orientation), strain, and dimensions (following the Scherrer equation [567]) of HAp crystals in the enamel [56], Figure 6a. One of the peaks in the diffraction pattern corresponding with the (002) plane (the diffraction peak) is widely used to analyse enamel texture, due to the location of this corresponding plane in the crystal lattice, being perpendicular to the c-axis of the crystal structure of the hydroxyapatite (HAp). Using motorised stages, data acquisition can be performed from the analysis of a single location, a line, a map, and finally in three dimensions (3D). This analysis can probe various sample thicknesses with a high resolution, which cannot be achieved using other conventional laboratory techniques.

Diffraction analysis provides details on the crystallographic orientation of enamel crystallites and describes their organisation within the sample, to understand any alteration of the organisation, either during or after being subjected to external conditions. The texture of enamel was initially characterised in 2007 using synchrotron, to reveal the orientation of the crystals in the enamel from the outer enamel surface to the DEJ, with a spatial resolution of 150 µm [89,506], Figure 6a. Characterisation of carious, remineralised, and demineralised enamel [172] showed that there was a loss in the texture in caries along the depth of enamel over a 500 µm thickness. Demineralised enamel exposed to salivary proteins was also studied [328]. Remineralisation studies have been performed in bovine enamel, where WAXS analysis was carried out after treatment of fluoride-containing dentifrices and using the analysis of the (001) peak as a function of depth [568], for the characterisation of fluorapatite (where a shift in the diffraction pattern observed) [569]; and the study of the biomimetic composite crystal structure [570] and the reflection and intensity of the diffraction peaks of the biomimetic materials compared to reference hydroxyapatite suggested the formation of crystals along the c axis.

WAXS experiments have been used to study human enamel at different stages of mineralisation with analysis of the texture of HAp crystallites in various regions of the enamel and observation of the presence of two orientation populations [378]. WAXS was also used for the study of crystallographic texture in the enamel of diseases other than caries: *ENAM*-mutated enamel was found with a lower texture compared to normal enamel [399]; in mucopolysaccharidosis, where crystallites were less oriented than in normal enamel [403,463]; and erythroblastosis fetalis (texture and size of the crystallites) [414]. In a comparative study of a human tooth and animal rostrum in a whale, more texture was present in human enamel, suggesting a different biomineralisation process [266]. Besides phase and texture analyses, WAXS can also be used to study the mechanical properties of materials, such as the measurement of the crystallographic strain of HAp before and after resin-based composite photo-polymerisation [380] with the determination of the evolution of lattice parameters in enamel. 

**Figure 7:**
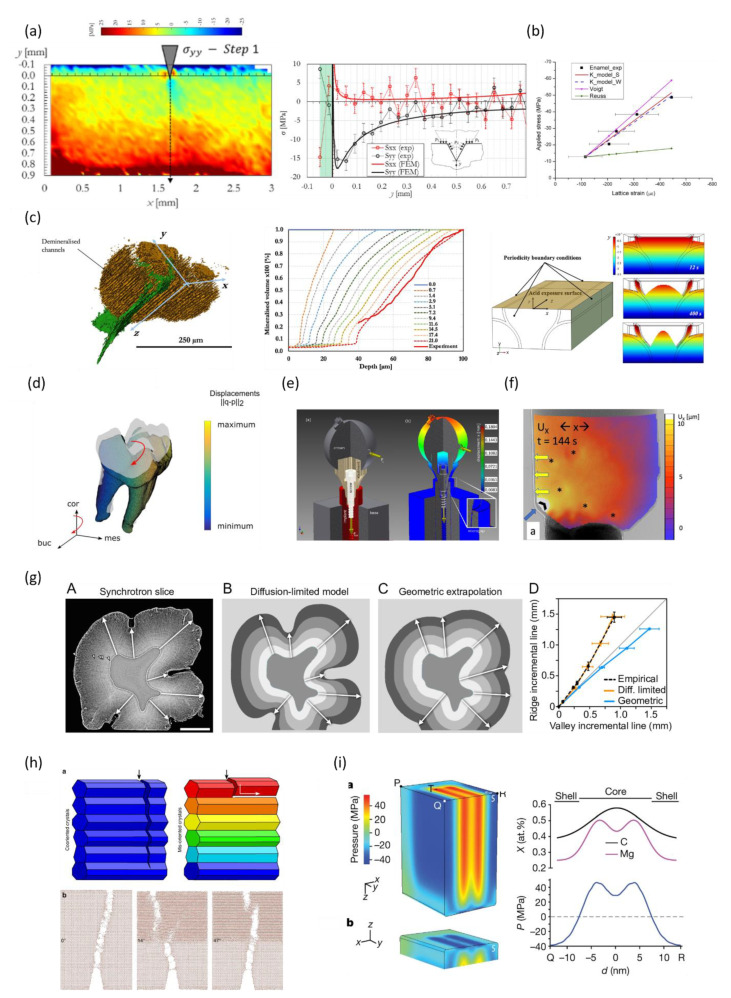
Application of modelling, simulation, finite element analysis, and molecular dynamics based on synchrotron experiments in dental research. (**a**) Analysis of the crack tip stress field in human enamel from WAXS data and the plot of the stress as a function of position (adapted from [349] with permission, Trans Tech Publications; Trans Tech Publications Ltd.). (**b**) Modelling results showing applied stress as a function of lattice strain in enamel, and in comparison to WAXS and SAXS datasets (adapted from [442], license https://creativecommons.org/licenses/by/3.0/ accessed on 30 March 2023). (**c**) Synchrotron tomography data of demineralised human enamel used to obtain the demineralised volume and geometrical model of enamel with finite element modelling of the demineralised front (adapted from [327], license https://creativecommons.org/licenses/by/4.0/ accessed on 30 March 2023). (**d**) Tomography of primate tooth and evaluation of the tooth displacement from unloading to loading (adapted from [597] with permission. Copyright 2021, Elsevier). (**e**) Finite element application showing the displacement of implant system from phase-contrast radiography of a dental implant (modified from [598], license https://creativecommons.org/licenses/by/4.0/ accessed on 30 March 2023). (**f**) Radioscopy carried out on a human root restoration, for a digital image correlation analysis of displacement and strain (adapted from [318] with permission. Copyright 2022, Elsevier). (**g**) Comparison of the incremental lines in pig enamel from synchrotron dataset and from simulations (adapted from Häkkinen T.J. et al. [364], license https://creativecommons.org/licenses/by/4.0/ accessed on 30 March 2023). (**h**) Schematic and molecular dynamic application of the crack propagation in different crystal misorientations based on PIC mapping dataset on human enamel (adapted from [77], license http://creativecommons.org/licenses/by/4.0/ accessed on 30 March 2023). (**i**) Finite-element modelling of the residual stress in enamel crystallite and the details of the pressure and composition of Mg and C (adapted from [193] with permission. Copyright 2020, Springer Nature).

One advantage of X-ray synchrotron-based WAXS is the possibility of in situ experiments, to study dynamic processes during mechanical tests, as well as thermal treatments, in liquid environments, with large samples, fast acquisition times, and a high resolution, which are not achievable using the other traditional laboratory techniques. Several studies, such as residual stress in enamel cracks under different loadings [349], were modelled using finite element analysis, to study the stress distribution in enamel (Figure 7a); compressive stress on dentine [441] and enamel [442]; to a model prediction from the hierarchical structure of enamel and determine the strains in the material (Figure 7b), as well as uniaxial compressive loadings [429]. The incorporation of in situ mechanical tests with synchrotron diffraction data acquisition was also performed in animal dentine (bovine, pig) under pressure, to better understand the toughness and elastic strains [571,572,573,574,575]. Additionally, the structural changes of human enamel and dentine during heating were also investigated in situ [425], where the d spacing increased with temperature and decreased back to almost the initial value after cooling, as well the effects of heat on dentine [435] for forensic science applications (Figure 6a). Heating and hydration studies were also carried out on dentine, to analyse the evolution of strain as a function of temperature. The strain (c-lattice parameter) was varied from −0.15% to 0% with an increase in temperature to 250 °C but remained stable until 125 °C [420], which is thought to be due to damage of collagen. 

Synchrotron X-ray diffraction has recently been used to study the structural changes during artificial demineralisation at a resolution of 150 µm [170], probing the evolution of demineralisation at multiple time points, to evaluate the evolution of the modifications occurring in the sample. The major advantages of this approach were the ability to track alterations of d spacing (Figure 6a), the length of the crystallites, and the intensity of the (002) diffraction peak at several locations from the maps acquired at different periods (Figure 6a).

One current key development in this area is the improvement of the spatial resolution of diffraction studies at nanoscale. In three recent studies on human enamel, the scan step and resolution were down to 500 nm, to investigate untreated and artificially etched enamel [56,354,563] (Figure 6a). Such a low resolution was obtained using Kirkpatrick–Baez (KB) mirrors [56,576]. In a previous study on a human tooth with erythroblastosis fetalis, the reported beam size during WAXS analysis was 0.53 × 0.39 µm^2^ [2]; however, a step of 2.6 µm, higher than previous studies was used [414]. A higher resolution was used to study archaeological samples of human cementum and dentine at 250 nm [310], and 200 nm and 120 nm for bone [577,578]. To date, the use of high-resolution diffraction analysis in dental research has not been widely applied. Probing carious enamel at nano-scale can reveal detailed alterations caused by the action of the acid produced in biofilm.

In addition to the improvement in spatial resolution, the combination of diffraction with other techniques that enable correlative analysis, such as spectroscopy, has been reported for cementum (using a resolution of 250 nm) [310], as well as the incorporation of tomography for bone [577,578,579]. In a study on caries, multi-dimensional WAXS analysis correlated with tomography [448] (at a resolution of 150 µm) revealed details of the lattice parameter and nanocrystallite size and suggested amorphisation of the crystal structure (Figure 6a). Similarly, preliminary work on enamel lamella was recently reported with microdiffraction tomography [353], as well as for dentine with nanotomography at a resolution of 120 nm [420] (Figure 6a).

Synchrotron XRD analysis has also been performed on carious and normal enamel. Generally, larger crystallite sizes and a higher texture index (calculated from the ratio of the (211) and (300) diffraction peaks) were found in normal enamel [171,434], indicating alteration of crystals in caries. WAXS has been used to analyse enamel fluorosis, revealing similarities in the diffraction pattern of HAp [490]. Another modality of the diffraction analysis is grazing-incidence synchrotron radiation diffraction [516,532,534], where the sample is placed at varying angles from 0.2 to 10°, as opposed to the standard transmission WAXS analysis. Using this approach, adult and temporary teeth were analysed, revealing larger crystal sizes in the latter. This technique was also used to compare enamel with and without laser irradiation treatment, as a caries prevention method, and it was suggested that one or several minor phases were formed due to the effect of the irradiation [489], as well as causing an increase in crystal size (suggested to be a consequence of the heating) [474].

In addition to WAXS, small-angle X-ray scattering (SAXS) was also applied for structural characterisation [580] in the study of dental tissues [453]. In comparison to WAXS, SAXS measures a larger distance of the crystallographic structure of the tooth; for additional details on the use of this technique, the reader is referred to the work of Guinier and Fournet [581]. The analysis of the SAXS pattern (Figure 6b) provides details on the texture, orientation (Equation (2) [173], repetition of the structure [582], porosity, degree of alignment, and a dimension parameter referred to as mean thickness (based on the Porod’s Law) [547] or T-parameter [480,583,584,585]. The following formula

(2) $$I\left(\phi \right)=I_{0}+I_{2}\cos(\phi +\varphi )$$

can be used for the analysis of the plot of intensity as a function of angular position ϕ, linked to the momentum transfer q ($q=\frac{4\pi }{d}$ with d periodicity) [173], after fitting with the cosine function, leading to the determination of the mean orientation from the phase φ (Figure 6b). The anisotropy of the material is determined from the full width at half maximum (FWHM) of the plot. Bone has been extensively characterised using this technique. For an interpretation of SAXS analysis, the reader is directed to Wagermaier W. et al. [582], who covered both WAXS and SAXS. The earliest study on human teeth was carried out on dentine, to study the region from pulp to DEJ, as well as comparing normal dentine and that affected by dentinogenesis imperfecta type II, where crystallite thickness and crystallite shape were calculated [547,548]. Differentiation of the region of enamel and dentine was seen, based on the respective orientations of the nanostructure [121]. Samples of dentine after demineralisation with EDTA, acetic acid, and lactic acid were also characterised using a scattering curve, to provide details about the particle shape of minerals [410]. For natural demineralisation, caries were investigated and enamel and dentine revealed different scattering intensities, with a decrease of the SAXS transmission data in the carious region identified using a beam size of 8 × 25 µm^2^ [472], Figure 6b. Another analysis using a beam size of 25 × 8 µm^2^ mapped enamel and dentine, to obtain the scattering orientation over various momentum transfer q ranges (such as 100 to 110 nm) (Figure 6b), and it was found that the orientation of the structure in carious enamel was close to the orientation of normal enamel [173]. In another study, the scattering intensity was found to be higher in carious enamel, and alternation of low and high alternation intensities, and the Hunter Schreger bands were visualised [457] with a beam size of 5 × 25 µm^2^. A similar technique was used to study natural and artificially demineralised regions of a molar using analysis of the anisotropy of periodicities between 40 and 150 nm [411] and a beam size of 30 × 10 µm^2^. Another study on dentine revealed a higher T-parameter in the carious region compared to the non-carious regions [480] (beam diameter of 30 µm), which suggested either reprecipitation or dissolution of small particles, Figure 6b. Enamel and dentine in burnt samples were also characterised in a post heat treatment [426,435,440] (beam size down to 14.5 × 19 µm^2^), and a decrease in alignment and increase in mean thickness were found with the increase in temperature, Figure 6b. Remineralisation of bovine enamel has been studied with SAXS, and the intensity of one specific peak (100) from WAXS/SAXS pattern was analysed [568]. Intensity and d-spacing for periodicity were studied in dentine, demineralised with EDTA or mineralised dentine [306]. The treated samples were reported to have a less intense integrated collagen peak (beam size 20 × 4 µm^2^).

In synchrotron experiments, in situ analyses can be performed, e.g., in situ SAXS to study the structure of enamel for archaeological studies and forensic science. Heat-treated samples were analysed, to reveal the evolution of the degree of alignment and the thickness of HAp crystallites, as a function of temperature during heating and cooling [425] (Figure 6b). The variation of SAXS patterns was also analysed during mechanical tests on enamel and dentine [441,442], as well as on bovine tissue under compression, together with WAXS measurement [586]. An in situ study on enamel exposed to lactic acid examined the rate of enamel demineralisation from SAXS peak intensities [170], and in combination with WAXS data, a schematic of the progression of the dissolution of enamel was suggested. Based on the SAXS data acquired with a spot size of 150 × 150 μm^2^, the evolution of the preferred orientation, dimension of the crystallites, and SAXS intensities were analysed over time (Figure 6b). SAXS, in combination with diffraction and spectroscopy, [587] can be used to study the biomineralisation process of HAp and for the development of new strategies to remineralise enamel, and to correlate this with other in situ analyses, for example in situ TEM imaging [588].

Finally, 3D SAXS analysis has been used to investigate the collagen fibres in dentine using X-ray sources [400,416,480]. One of the main difficulties of this new development lies in the processing and analysis of the data acquired, which requires algorithms for reconstruction, long acquisition times, and a large amount of computer memory [589,590,591]. Recently, in a proof of concept study for the 3D reconstruction of the SAXS signal, small angle X-ray scattering tensor tomography from dentine samples provided the internal distribution of the orientation of the collagen fibres over large regions [329,400,416] (Figure 6b). This method has also been used to study the orientation of the crystal structure in bone [578,592,593]. Laboratory X-ray tensor tomography has been carried out to provide details of the orientation of human dentinal tubules [594], as well as enamel and dentine [595]. SAXS tensor tomography can be complemented with diffraction tensor tomography; for example, to study bone [596].

### 3.2. X-ray Imaging Techniques—Tomography, Ptychography and ‘Rich’ Tomography

X-ray microcomputed tomography and imaging techniques [599,600,601] have been extensively used in studying tooth structure, due to their less destructive analysis and the possibility of analysing samples in two dimensions (2D) (radiography), 3D (volume), and 4D (i.e., 3D with the addition of time). Chemical composition has also been studied in other applications, adding a further dimension, for instance in bone [577].

**Figure 8:**
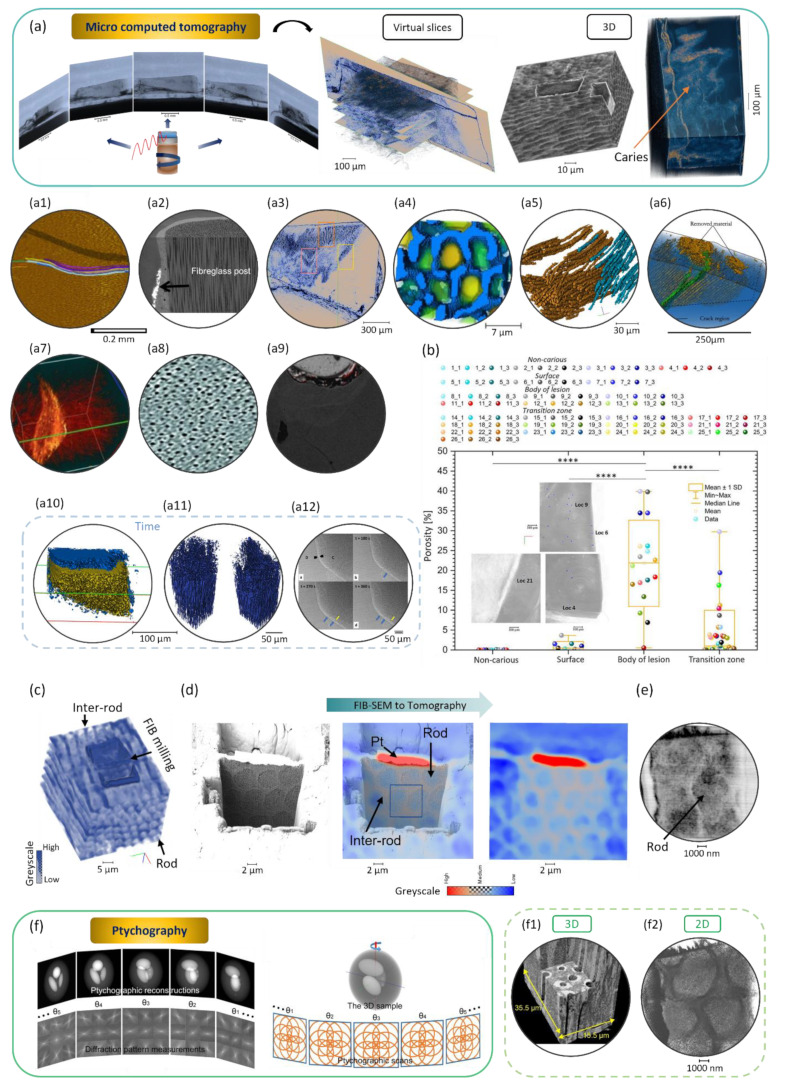
Details of tomography and ptychography application in dental research. (**a**) Illustration of the tomography setup with rotation of the sample and the acquisition of projections, which after reconstruction produced a 3D dataset, adapted from [39], license https://creativecommons.org/licenses/by/4.0/ accessed on 30 March 2023. Details of some application (**a1**) pathways of enamel rods in a Neanderthal molar (adapted from [451] with permission. Copyright 2012, Elsevier). (**a2**) Three-dimensional rendering of a restored tooth (adapted from [345] with permission. Copyright 2020, John Wiley and Sons). (**a3**) Virtual slice from human carious enamel with a voxel size of 0.325 µm (adapted from [39], license https://creativecommons.org/licenses/by/4.0/ accessed on 30 March 2023). (**a4**) Rod and inter-rod visualisation in 3D (adapted from [57] with permission. Copyright 2021, Elsevier). (**a5**) Pathways of the rods from a carious enamel (adapted from [39], license https://creativecommons.org/licenses/by/4.0/ accessed on 30 March 2023). (**a6**) Three-dimensional rendering of artificially demineralised enamel and cracking (adapted from [327], license https://creativecommons.org/licenses/by/4.0/ accessed on 30 March 2023). (**a7**) Three-dimensional rendering of human dentine and cracking (adapted from [359] with permission. Copyright 2020, Springer Nature). (**a8**) Reconstructed tomogram of human dentine (adapted from [357] reproduced with permission of the International Union of Crystallography). (**a9**) KES image based on mercury analysis (adapted from [376] with permission. Copyright 2018, Oxford University Press) and application in situ. (**a10**) Three-dimensional rendering of demineralised enamel at two time points (adapted from [304]) and (**a11**) 3D rendering of demineralised dentine at two time points (adapted from [333] with permission. Copyright 2021, Elsevier) and (**a12**) radiographs following polymerisation and at different times (adapted from [318] with permission. Copyright 2022, Elsevier). (**b**) Plot of the porosity in enamel as a function of locations in a carious tooth from tomography analysis, adapted from [39], license https://creativecommons.org/licenses/by/4.0/. (**c**) Three-dimensional rendering of carious enamel with the location of a FIB milled cross-section, which could be used for further analysis, such as nanoprobe, TEM (adapted from [39], license https://creativecommons.org/licenses/by/4.0/ accessed on 30 March 2023). (**d**) Correlative image analysis with SEM image and tomography virtual slice from demineralised enamel in the carious region (adapted from [39], license https://creativecommons.org/licenses/by/4.0/ accessed on 30 March 2023). (**e**) Differential phase-contrast (DPC) image of carious enamel, in a similar location as the image in (f2) (adapted from [78], license https://creativecommons.org/licenses/by/4.0/ accessed on 30 March 2023). (**f**) Details of the 3D ptychography principle, adapted from [611], license http://creativecommons.org/licenses/by/4.0/ accessed on 30 March 2023, and illustration of application. (**f1**) Three-dimensional rendering of human dentine with a pixel size of 65 nm (adapted from [413], https://creativecommons.org/licenses/by/4.0/ accessed on 30 March 2023) and (**f2**) 2D reconstruction of the transmission data from ptychography acquisition carried out on carious enamel with visualisation of the differences in structure in the rods and inter-rods (adapted from [78], license https://creativecommons.org/licenses/by/4.0/ accessed on 30 March 2023). For additional details on the scale of the images, see the references.

One of the earliest publications on synchrotron microradiography of human dental tissue was that of Takagi et al. [303], which identified demineralised enamel, the more mineralised surface zone superficial to the body of the lesion, and characterised dentinal tubules and striae of Retzius, reporting a significant improvement in the observational details in comparison with conventional microradiography. This has been extended to 3D imaging (tomography), by acquiring projections at a series of angles and reconstructing the volume from those projections [600,602] (Figure 8a). Tomography can be performed using various acquisition techniques [603], e.g., absorption or phase-contrast enhanced. The effect of the phase-contrast was illustrated in a study of dentine that visualised the difference in contrast as a function of the distance between the sample and the detector [458].

X-ray tomography is based on the principle of attenuation contrast described by Lambert–Beer’s law [604], in the form of a mono-energetic X-ray $I_{mono}$ (Equation (3)):

(3) $$I_{mono}\left(E\right)=I_{0}\left(E\right)e^{-\int_{0}^{s}\mu \left(\rho ,Z,E\right)ds}$$

where E indicates the energy, Z is the atomic number, ρ is the density, e is the elementary charge, s is the thickness, μ is the attenuation, and $I_{0}$ is the transmitted X-ray intensity.

In contrast, phase-contrast X-ray tomography is based on capturing the phase shift taking place between features of the sample and provides details of where the density variation between the features is low. 

X-ray microcomputed tomography provides major advantages, as it can be used to analyse fragile samples, samples (or cracks) surrounded by a different material, as well as in various experimental environments and setups, such as in mechanical tests, conserving a high resolution and high signal-to-noise ratio. The results from tomography can be used to determine structural details, as well as densitometrical measurements; however, the latter requires the use of a calibrant and ideally monochromatic X-ray beam source. In dental research, samples from modern and fossil records of human teeth have been analysed to gather insights into their internal dental structure. An example of the internal output of this technique can be found in the investigation of chimpanzee and Neanderthal molars using the segmentation and extraction of the 3D structure of rods from the enamel surface to DEJ with a voxel size of 0.678 µm [451] (Figure 8a). This technique has also been used in several other fields of dentistry, to study endodontic post and apical sealers, with the analysis of defects with a pixel size of 5 µm [526]; to study fluorosis, with the analysis of X-ray absorption coefficient in comparison to a healthy tooth with a voxel size of 12.94 µm [484]; chemo-mechanical treatments for root canal fillings, with analysis of tissues and debris [371]; as well as cracks formed after root canal preparation, extraction, and cryopreservation [342,417,456]. Similarly, X-ray tomography was also used to investigate post-endodontic restorations, to assess the bonding of composite resin cement, and identified gaps and voids with a pixel size of 650 nm [344,345] (Figure 8a), as well as remineralisation [314]. Virtual slices from tomography (referred to as virtual histology) were also recently compared with histological descriptions visualised using optical microscopy, with a higher resolution from the synchrotron data (effective pixel size of 0.9 µm instead of 2 µm) showing that optical images were sufficient to characterise the daily secretion rate of enamel [309]. In terms of structure and tissue, additional details of application research which has been done are summarised in Table 1.

Synchrotron radiation tomography for the analysis of human dental tissues was reported on a carious sample in the 1990s using X-ray tomographic microscopy (XMT), with feasibility for analysing hard tissue using tomography as a proof-of-principle on a tooth (without the remainder of enamel), together with studies of X-ray attenuation [556,557] and on a dentine sample with loss of enamel due to caries, opening the possibility of imaging the lesion and the tubules [555]. 

In samples with a remainder of enamel, the first publication related to enamel tissue was reported in 2004, which analysed normal and carious enamel with virtual slices and 3D rendering from synchrotron tomography analysis, with a voxel size of 1.9 µm. Using this technique, the lesion was observed, as well as the striae of Retzius, with the details of the mineral concentration showing a visualisation of the surface layer more mineralised than the lesion [175]. A higher resolution was obtained for the study of caries, and reconstructed data were obtained with a voxel size of 325 nm on different regions of carious enamel (advanced enamel caries and less advanced early enamel caries), as well as enamel with artificial demineralisation [39,57,319,327] (for more details, see Besnard et al. [57]). In addition to structural visualisation, some parameters were measured from the tomography data, such as the thickness of the lesion, Euclidian distance between voids, and Feret’s diameter of the lesion, adding details about the characterisation of carious enamel, see [39,57] for additional details. Tomography provided details of the tissue structure, which allowed comparison of different samples preliminary identified as advanced enamel caries and early enamel caries [57]. The latter carious studies revealed a rod and inter-rod 3D structure at sub-micron resolution over a large volume with visualisation of the pathways of demineralisation in 3D [39,319], to compare with other imaging modes, such as optical and conventional X-ray tomography. The demineralisation of enamel was tracked within the sample, without the need for sectioning the material (less destructive) and quantified the demineralised regions in 3D. The study combined high resolution and contrast, to distinguish different regions of the enamel. Analyses were also done with a lower resolution and pixel size than 325 nm [39], such as on cavitated caries lesions with a pixel size of 1.3 µm [346,385] and possible root caries with a voxel size of 615 nm [361]. In another study related to carious enamel, enamel slabs were placed intraorally using intro oral appliances, to mimic the conditions of natural demineralisation, and the spatial resolution was ~7–8 µm and the carious acid-attack on the sample and cavities were analysed, together with the importance of knowledge of individual factors [446]. In terms of resolution, higher resolutions in the study of dental tissues are reported and summarised in Table 2, with a few studies on another biological sample, bone.

In comparison with conventional X-ray tomography devices, the advantages of synchrotron X-ray tomography include its capability to combine a high-resolution, fast acquisition, range of energies, small and large volume samples, and localised region [57]. Using localisation of the regions of interest in a volume of material, the porosity of carious enamel was investigated, with significant differences found between the body of the lesion and other zones in the sample in the measurement analysis [39], Figure 8b. Multi-scale correlative imaging with tomography was reported in a previous study that combined tomographic imaging with FIB-SEM, Figure 8c,d. One advantage of the analysis using a synchrotron was the possibility of varying the energy of the X-ray beam, to analyse mercury from amalgam restorations that diffused into the tooth tissue using the so-called K-edge subtraction (KES) technique, which is based on the characteristics of the K-edge energy of mercury. Acquisition of a tomogram below and above this energy, followed by the subtraction of the two data sets, enhanced the contrast of the region containing mercury [376] (Figure 8a). In addition to mercury, elements such as Sn and Ag were also characterised [545]. Due to the possibility of combining several detectors in a synchrotron beamline, tomography has also been employed with other techniques, such as SAXS, and was used for the correlative analysis of carious teeth [457,472], as well as normal dental tissue [121].

Tomography and radiography analysis generally produces rich and large datasets, which can be used for further simulation studies (Figure 7c–f) and in combination with WAXS data (Figure 8f). Three-dimensional datasets can be used for further analysis such as in modelling, finite element, mesh, and dental education [612]. Extracted information from tomography has been used as input for a model to study artificial demineralisation of enamel [327], with the simulation of the demineralisation of enamel at different times, Figure 7c. Tomography datasets were used to develop a simulation of the enamel matrix secretion from a diffusion-limited model on a pig tooth, using the determination of the DEJ region, Figure 7g. This model was also applied to human and primate enamel, with similar results as in the determination of the enamel morphology from DEJ [364]. 

The previous approaches were mainly based on the analysis of dental tissue samples in air and under static conditions. Given the flexibility of tomography techniques and the information provided internally from the material, in situ techniques have been employed to study real-time details of processes in various environments (time-resolved techniques). This type of research experiment is designed to probe instant reactions, as and when they occur, to determine rate variations and to visualise the history of the processes, which are otherwise lost in static experiments. In early studies, dentine demineralisation was investigated using time-lapse tomography [552,553], as well as with conventional radiography of enamel exposed to acidic solutions [613]. The combination of a fast acquisition time over large sample dimensions, with high resolution, as well as the ability to use environment chambers and mechanical tests, cannot be matched by other techniques. Such approaches have been used in several other fields, e.g., steel, alloy, electrolyte samples, magmatic processes, and self-healing materials [614,615,616,617,618,619]. In situ experiments have been carried out on enamel demineralisation for the calculation of the rate of modification of the tissue structure and visualisation with time [304], allowing time-lapse analysis of rod and inter-rod demineralisation (Figure 8a), in contrast to previous studies using different pH solutions and single time points [57], using intraoral mandibular appliances [446], or artificial demineralisation with time using laboratory tomography and radiography [145,620]. In situ analysis provided a visualisation of the anisotropy of the diffusion of the acid in the enamel structure [304]. Similarly, the demineralisation of human dentine was also studied, and the evolution of the diameter of the dentinal tubules was extracted and measured over time at a pixel size of 325 nm [333] (Figure 8a).

In addition to the study of demineralisation, the use of time-resolved tomography can further aid in the investigation of remineralisation strategies and structural arrangement, such as in studies where the mineralisation was visualised on enamel after treatment with a polymer [368]. Another remineralisation study on human dentine treated with fluoride used synchrotron tomography at a voxel size of 51 nm [412] over a small region. The porosities were visualised in the demineralised zones, and a difference in the response to the remineralisation was found between the tubular and inter-tubular regions, with an increase of linear attenuation coefficient detected in the inter-tubular region. Studies also analysed the precipitate from silver diamine nitrate and fluoride, with analysis of the contrast obtained after the acquisition, and radio dense zones were visualised in comparison to the control sample at a resolution of 1.44 µm, suggesting remineralisation [305,621] using synchrotron and laboratory tomography. The polymerisation of dental composites in teeth was also studied using in situ phase contrast-enhanced radioscopy, providing visual information on the delamination occurring during the polymerisation, with the formation of a gap between the adhesive layer and the dentine with time, Figure 8a. The delamination was also visualised in 3D in a static experiment, to obtain the internal structure [318]. Rapid data acquisition is essential to study fast processes, as is evident from these studies; and considering the structural features of enamel, ranging from macro to nano scale, a high resolution is required to visualise the range of sub-micron features in these tissues. The fast acquisition and flexibility of tomography and radiography could not only be carried forward to study biomineralisation and amelogenesis, but also remineralisation and dental restorations using different materials.

Another synchrotron imaging modality referred to as directional X-ray dark-field imaging (DDFI) [622] provides two imaging modalities: radiography (transmission image) and a dark field image. This was utilised to obtain details of the variation in the orientation of the structure of human dentine and enamel [481]. Grating-based X-ray phase tomography was also used to study tissues. The samples were human teeth and a pig tooth, with measurements of the mass density related to the refractive index, which improved the sensitivity of the analysis in comparison to X-ray absorption microtomography [407]. Dark-field tomography has recently been used to study cracks in teeth with and without caries. The images obtained showed a correlation of the attenuation of the X-ray in the sample and the small angle X-ray scattering [623]. Another imaging mode is differential phase-contrast (DPC) imaging, which was applied on dentine in a carious tooth and correlated with XRF mapping [169], as well as on an unfilled tooth [483] and recently on human carious enamel lamella, showing the lesion from the variation in contrast, which was carried out with a beam size of 45 × 55 nm^2^ and a step of 50 nm, and correlated to other modalities (SEM, XRF and ptychography) [78], Figure 8e. This technique is based on the index of refraction of the materials imaged and can be beneficial in a biological sample where the absorption may be low [624,625]. Carious lesions were also detected using a different technique based on near-field technique, THz near-field imaging [523], revealing the lesion corresponding to the modification of contrast.

Tomography can be used for studies of mechanical properties, with the visualisation of modification in the 3D structure and propagation of the crack during indentation in static and in situ experiments [626,627,628,629]. Mechanical analysis has been applied in 2D, using digital image correlation on radiography images with time to characterise changes during the tooth restoration [318]. Mechanical experiments using synchrotron radiography can be extended into 3D with digital volume correlation, as performed on bone [629]. 

Similar to diffraction analysis, there is a need to improve the resolution to reveal details that could not be visualised previously. Studies have been performed using a transmission X-ray microscope [630] and using a technique moving beyond the limitation of the optics in 2D and in 3D [631]. The latter requires retrieving the sample’s phase and the amplitude of sample using a technique referred to as ptychography [632] without a lens [633,634], and which can be carried out in 2D, 3D, and 4D [635] and has been used in various fields using X-ray or electron microscopy [292,636]. In dental research, the technique is limited [78,413,637,638] and X-ray ptychography (Figure 8f) has made it possible to characterise human dentine (energy of 6.2 keV) with a spatial resolution of 158 nm [413] using 3D ptychography and recently to map carious enamel from a lamella with soft X-ray (energy of 1 keV and reconstruction performed with a pixel size of 8 nm) [78]. In dentine, this provided a localised variation of the composition with high spatial resolution and the visualisation of branches in the dentine and the variation of density in the ITD matrix [413], Figure 8f. In enamel, with the resolution obtained, it was possible to visualise the distribution of crystallite in rods and inter-rods and to reveal the clear variation of orientation in these two regions, Figure 8f. The thickness of the sample was ~2.4 µm higher than the usual TEM lamella and with the advantages of reducing the effect of the FIB milling on the dataset acquired, and also with the potential to be carried out in 3D [78]. The sample analysed was also characterised by X-ray fluorescence spectroscopy, leading to an innovative correlative nano-analysis of carious enamel down to nanoscale, with information of composition and structure [78] (Figure 2d), and showing the importance of nanoscale in the analysis of enamel. Ptychography is not limited to imaging and the qualitative detail of the sample but can also be used as a quantitative technique to determine the mass density of the sample; however, this requires some knowledge of the sample and energy used [639] and has been carried out on human dentine [413].

The internal structure of teeth (which can be observed using tomography analysis, as described earlier) can be further exploited for correlation analysis with other imaging modalities. For example, the WAXS technique has the limitation of a lack of detail about the volume probed and a direct correlation of the localised regions between different modalities. Recently a new setup has been developed to allow direct correlation from the WAXS location and projection data, which can then be extended to 3D datasets [640] using two beams. The precise correlation between the detected signal and its corresponding location in the volume probed is essential and can be used in static and dynamic setups. In parallel, the signal from the samples after exposure to synchrotron X-ray radiation can be correlated with other imaging modes, such as neutron imaging, providing additional details based on the origin of the signal from the cross-section of the elements [641,642]. This was applied to the analysis of a fossil orangutan molar, and an increased contrast was found in the tissues in comparison to X-ray imaging [641]. Hence, the tomography technique represents a useful tool for providing rich 3D detail about samples, which can be implemented using various imaging techniques.

### 3.3. Spectroscopy—XRF, XANES, PIC Mapping, and FTIR

This section describes a range of synchrotron spectroscopy techniques for the analysis of chemical and local environment elemental analysis, such as using X-ray fluorescence (XRF), X-ray absorption near edge structure (XANES), X-ray absorption spectroscopy (XAS), and Fourier transform infrared spectroscopy (FTIR). For additional details on spectroscopy, the readers are directed to [643,644,645,646,647,648,649].

**Figure 9:**
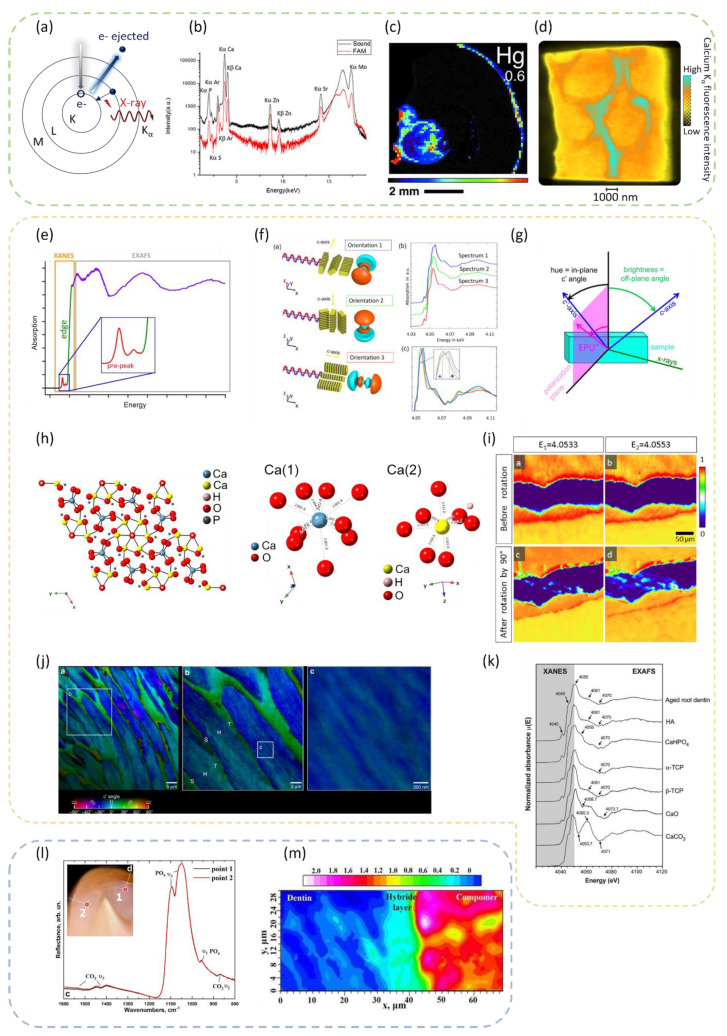
Details of the spectroscopy techniques used for dental research applications. (**a**) Principle of XRF analysis based on the emission of the characteristic X-ray (e- refers to electrons, adapted from [651], license https://creativecommons.org/licenses/by/4.0/ accessed on 30 March 2023). (**b**) XRF spectra of sound enamel and enamel from a patient with FAM20A mutations, (adapted from Lignon G. et al. [652], license https://creativecommons.org/licenses/by/4.0/ accessed on 30 March 2023). (**c**) XRF map of a tooth with caries with the spatial distribution of Ca from a 100 µm thick slice (reproduced from [499] with permission of the International Union of Crystallography). (**d**) XRF map of the Ca K_α_ fluorescence intensity acquired on human carious lamella with a beam size of 45 × 55 nm^2^ (adapted from [78], license https://creativecommons.org/licenses/by/4.0/ accessed on 30 March 2023). (**e**) Illustration of the XANES and EXAFS region (adapted from [653] with permission. Copyright 2015, Elsevier). (**f**) Schematic and plot of the XANES spectra at the Ca K-edge under different polarisations, to illustrate the principle of X-ray linear dichroism in the tooth (adapted with permission from [363], © The Optical Society). (**g**) Illustration of the PIC mapping analysis (adapted from [654] with permission. Copyright 2018, Oxford University Press). (**h**) The crystal lattice of HAp and the details for the two Ca sites, obtained from CrystalMaker^®^ (CrystalMaker Software Ltd., Oxford, England) using cif file 203,027 Inorganic Crystal Structure Database (ICSD) [655,656] (adapted from [657] with permission. Copyright 2011, Elsevier). (**i**) Polarization XRF maps of dentine and enamel at Ca K-edge with two energies and with rotation of the sample (adapted with permission from [363] © The Optical Society). (**j**) PIC mapping of human enamel from the image at ±0.2 eV of the Ca L-edge, showing the crystal orientation (adapted from [77], license http://creativecommons.org/licenses/by/4.0/ accessed on 30 March 2023). (**k**) EXAFS spectrum of dentine in comparison to other samples (reproduced with permission from Kantrong N. et al. [314]). (**l**) FTIR spectra of a tooth with fluorosis with the details of the radical PO_4_^3−^ and CO_3_^2−^ (adapted from [348], license http://creativecommons.org/licenses/by/4.0/ accessed on 30 March 2023). (**m**) FTIR map of dentine and composite with the details of the intensity of the absorption band 1762–1701 cm^−1^ (adapted from Goloshchapov D. et al. [337] under license by IOP Publishing Ltd., under the terms of the Creative Commons Attribution 3.0 licence, license https://creativecommons.org/licenses/by/3.0/ accessed on 30 March 2023).

X-ray fluorescence spectroscopy using synchrotron sources has several advantages in comparison with the other standard laboratory analyses mentioned in [650]. An XRF analysis is based on the characteristic X-rays emitted by different elements [252], see Figure 9a. The emitted X-rays result in specific spectra at the analysed locations, Figure 9b. XRF has been used to characterise dental samples (with and without caries and samples affected by other diseases), to study various elements, such as Pb, Hg, Ca, P, Sr, Br, Zn, Cu, and Fe. Similarly to diffraction techniques, data can be acquired from either a point, a line, 2D maps, or 3D regions. The beam energy used for spectroscopy is an important parameter to be considered in samples analysis, as it needs to be high enough to excite the element of interest (to eject the orbital electron and in turn emit the characteristic X-ray), similarly to the traditional energy-dispersive X-ray spectroscopy carried out in SEM. For example, soft X-rays with an energy below 4 KeV cannot be used to study the K-edge of Ca in enamel.

The first reported study on spectroscopy using a synchrotron to study tooth enamel with XRF was published in 1986 [561]. The elemental analysis of human tooth enamel has been studied for several applications, such as analysis of trace elements in enamel, toxicity analysis or diffusion of mercury (Hg), quantitative elemental analysis of different locations of enamel samples, and the evaluation of dietary habits and environmental influences. The earliest studies that used the fluorescence technique (based on synchrotron-radiation induced X-ray emission SRIXE in comparison to laboratory fluorescence [658]) focused on the analysis of traces of lead (Pb), Ca, and zinc (Zn) elements [558,559,560]. As Pb is known to have neurotoxic and associated negative effects, it has been extensively studied [382,482,542,659]. Environmental contamination and dietary habits were also studied by analysing various elements [486,549,550]. Hg and other elements present in dental amalgam were investigated in restored teeth, to assess their diffusion and distribution, as Hg has a potentially hazardous effect at high concentrations [499], Figure 9c. Similarly, other hazardous elements were also investigated [499,527,536,542,550,551]. Chemical analysis using X-ray microprobe and micro X-ray fluorescence is suitable not only for enamel, but is also used for dental calculus [544,554,660], dental pulp tissue, and dentine (analysis of Ca, P, Zn) [315], as well as cementum (Ca and trace metals), which can help in determining the chronology of the exposure to these elements [509]. In another study, Ca, P, and Sr were analysed in enamel powder and tooth slices that were irradiated using a free electron laser. The study revealed a decrease in the elemental content in the irradiated area [546]. XRF measurements were also performed, to investigate disease and the modification of natural situations. Elemental concentration of dental tissues with and without renal insufficiency patients was studied [531], which showed a significantly high concentration of Pb found in the pulp in comparison to healthy people; as well as the study of the effect of cigarette smoke (contamination from heavy elements) on bovine teeth [661] and a high content of Pb and Cd found in enamel and dentine after smoke exposure in comparison to a control sample. In addition to these studies, a sample with carious lesion and amalgam was studied, and the diffusion of elements including Fe and Hg was identified [551]. Carious teeth and artificial demineralised tissue were also studied with the analysis of Ca, Zn, Cu, Pb, and Br [195]. In other studies, Cu and Zn were found to be in higher concentration in carious regions [169,538] compared with unaffected locations. One of the analyses provided 2D maps of the distribution of the investigated elements with a high resolution, down to 1 µm (1 × 0.5 µm^2^ of beam size) [169], Figure 9c. More recently, the resolution was lowered further in the study of a human tooth from a patient suffering from HELIX syndrome, where a beam size of 500 × 500 nm^2^ was used to show the variation of Sr in enamel and dentine, which was suggested to be from a renal dysfunction [308]. In another study, a resolution of 250 nm was used to analyse the variation of Ca and Zn intensity in human cementum and dentine [310]. A higher resolution than these studies was also used to characterise carious enamel and the preferential demineralisation occurring in a sample. XRF with a beam size of 45 × 55 nm^2^ and a step of 50 nm was performed, showing the distribution of Ca K_α_ fluorescence intensity in the lamella [78], Figure 9d. The intensity of Ca was found to be higher in the inter-rod region than the rods, which correlated with an SEM image of the sample as well as the variation of crystallite organisation seen with ptychography.

X-ray linear dichroism polarization-dependent imaging contrast (PIC) mapping is another type of analysis that can be performed using synchrotron beams, and it has been applied in the study of several biominerals, e.g., abalone nacre and coral [662,663] (additional details in SI—Table S2). This is mainly a surface technique that provides details about the orientation of crystals and which was successfully used on enamel, with significant details provided on the orientation of the crystallites at nanoresolution [77]. The technique is based on the dichroism of the apatite crystal, in accordance with the polarisation angle, and can be observed either from the various elements L-edge or K-edge (electron’s binding energy [651]) (Figure 9a) obtained from the XANES spectrum (Figure 9e) using a photoemission electron spectromicroscope (PEEM). The analysis can be carried out using XRF at several energies to reconstruct the XANES spectra, followed by comparison at different locations and rotation angles. Different XANES patterns are reported to correspond to different orientations of the crystal structure of HAp (Figure 9f), and then the orientation texture of the material can be retrieved from the processing of the dataset and mapped. For example, anti-correlated energies below and above the Ca L-edge (352.6 eV) were selected from each pixel imaged on tooth enamel and then divided, to create a PIC map from the different polarization angles using the Malus law [77,654]. For human enamel, this technique provided details of rods and inter-rod substances at different resolutions, from micro to nano scale [77,363]. It has been applied to the characterisation of samples based on the absorption edge of various elements present in samples, e.g., Ca, O, and C [77,372,654], on the polarization plane and c-axis, leading to the colour map encoding the details of the off-plane angle of c-axis and ‘c’-axis’ detailed in Figure 9g. It offers high-resolution scans at or below 50 nm. Specifically for Ca, the molecular environment of specific atoms was analysed at positions 1 and 2 in the crystal lattice [338], Figure 9h. PIC mapping, thus, has great potential for use in biomineralisation studies [663,664]. The technique, however, requires special sample preparation, as it is surface sensitive, especially at high resolutions, due to the origin of the signal from a depth of a few nanometres. In a study involving PIC mapping with a high-resolution and Ca L-edge [77], the sample was coated with 1 nm of Pt in the region of interest and surrounded by 40 nm of Pt, to carry out PEEM analysis [662]. In another study performed at submicrometer resolution (~0.6 × 0.8 µm^2^ beam size) with the Ca K-edge analysis, peritubular dentine and the rod texture in enamel were visualised, without the requirement for nanometer-polished surfaces [363], Figure 9i. In a high-resolution PIC mapping study [77], the Hunter–Schreger bands could be seen at low magnification. At high magnification, the orientation of the crystals (c-axis orientations) was visualised at nano-resolution using PIC maps, and a misorientation in the organisation of crystallites was identified, Figure 9j. Multiple orientations within rods over small regions and mis-orientation of some crystals was visualised. In general, the c-axes of the crystals were found to vary from 30 to 90° in the rods. That data were used for molecular dynamics analysis to study the toughening mechanism as a function of crystal orientation, and it was found that, with mis-orientation of the crystals, the cracks were deflected, Figure 7h. This toughening mechanism was also found in a biomineral using PIC mapping and molecular dynamics [665]. A PIC mapping technique was further used to compare the structure of human enamel with that in other species [322] and to correlate with the hardness measurement. Mis-orientation in the structure of the enamel of teeth plays an important role in its mechanical properties.

The XANES technique provides details about local elemental environments (coordination environment) and can be employed to characterise enamel and biocomposites for the development of biomimetic materials [338,666,667], as well as to compare them using their spectra, e.g., HAp, FAp [668]. P L-edge and Ca L-edge XANES measurements were investigated, to measure the Ca/P ratios in enamel and dentine, in comparison to composites [338]. The detection of elements and configuration is important in the development of new materials. The analysis of the XANES spectra also provided necessary information on Hg L-edge, for studying the Hg distribution in enamel obtained from teeth with an amalgam filling, which showed variations in spectrum with location [499]. Root dentine exposed to several demineralisation and remineralisation treatments were analysed at the Ca K-edge, and the chemical composition of dentine was suggested accordingly [314], Figure 9k.

At a higher energy than the XANES spectrum is the extended X-ray absorption of fine structure (EXAFS) [653,669], Figure 9e,k, which combined are referred to as XAFS [383]. The XAFS technique provides details of the electronic structure of samples. The signal in EXAFS arises from the local sample structure and is related to the surrounding atoms, from which the scattering of photoelectrons originates [670]. EXAFS has been carried out on dental tissues and dental calculus [193,660]. Human enamel was investigated, to analyse the Mg K-edge, and was compared with Mg ACP [92,193], revealing similarities in the enamel structure, as well as comparisons with rodent enamel, showing the presence of Mg in the crystallite cores. These results were combined with other nanocharacterisation techniques, such as APT and TEM, to model the amelogenesis process, as well as to provide detail about residual stress in enamel crystallites from chemical gradients [193,671], Figure 8i. It was suggested that residual stress leads to an increase in the solubility of crystallite cores compared to the surrounding, which was visualised as a preferential demineralisation of the crystallites. Hg was also studied, and the Hg L-edge analysis in locations of amalgam and the interface with dentine showed no significant differences in the coordination environment of Hg [522], and one mercury species was found [527]. EXAFS above the Ca K-edge could be used to analyse the distribution of atoms in HAp (for example, the first peak was assigned to the environment of six oxygen atoms and Ca-O interaction), and the (dis)order, in the enamel, the spectrum was compared with the known spectra from synthesised HAp or bone [672,673,674,675,676], as well as studying the formation of HAp and Ca-deficient HAp with and without XANES [677,678]. In another application, the demineralisation of bovine dentine was assessed by analysing the local environment of the Zn-K edge with both XANES and EXAFS [679]. Dentine replacement materials have been studied using the two techniques and the Ca K-edge [680]. Other elements were also analysed and Zn-doped dentine was investigated with K-edge, to study Zn effect on the acid resistance [31]. In studies on the treatment of caries, XAS Ca K-edge and Zn K-edge were also used to analyse glass ionomer cement synthesis for restoration purposes [681], as well as XAFS of Sr K-edge on enamel and dentine powder exposed to surface pre-reacted glass-ionomer [383]. As strontium is thought to improve the remineralisation and acid resistance of enamel, the Sr distribution was analysed and compared with a radial distribution function (including details for Sr-P and Sr-Ca). Preferred sites of demineralisation from the analysis of the absorption of XAS could be potentially determined [682], while the environment of elements Ca L-edge, Mn L-edge, Ca K-edge, and P K-edge could be analysed [682]. These analyses required comparison with a theoretical calculation of the total energy of Hap, in addition to the experimental work [682].

This technique can also be applied to investigate in situ processes [683]. The ability to probe chemical composition can be used to decipher the biomineralisation processes in combination with other techniques, as reported in studies of the chiton tooth and on Ca-phosphate [684,685]. In parallel with the analysis of Ca K-edge with XANES, resonant Raman scattering (RRS) was applied on enamel and dentine with an energy of 88.5 eV below the Ca K-edge, and the characteristic spectra enabled the different tissues to be distinguished [445].

Three-dimensional spectroscopy analysis has been implemented in synchrotron studies; however, its application has been limited in dental research. PIC with ptychography was carried out on coral, to provide 3D details of the orientation of the crystals [635], and X-ray fluorescence computed tomography applied to bone reported the distribution of Ca and Ga K-edge [577]. Confocal XRF measurements have been performed at different depths in a restored tooth, where the distribution of Zn was visualised, suggesting that diffusion had occurred into the dentine [686]. It could be envisaged that performing 3D XANES enabled additional details of biomineral materials to be obtained with association with X-ray spectro-tomography, which has been recently used for cathode particles [687]. Another major development is the improvement of spatial resolution to acquire XRF-XANES results at nanoscale (e.g., bone with a beam size of 32.3 × 30.5 nm^2^ [577]). To enrich the nano-spectroscopy dataset, another modal analysis was implemented on the same locations with simultaneous acquisition, e.g., diffraction analysis was applied for animal dentine [688,689], human bone [577], and differential phase contrast on human carious enamel [78].

The previously mentioned spectroscopy techniques are based on X-ray absorption in the sample from elements, in contrast to vibration spectroscopy of functional groups, such as in FTIR [690]. From this perspective, it is often reported that FTIR produces patterns that represent fingerprints of the molecular chemistry of materials. Synchrotron FTIR offers advantages over conventional laboratory FTIR equipment [691], including high signal-to-noise ratio and high brightness, and it was used to analyse enamel fluorosis from the carbonate and phosphate groups, to reveal carbonate substitution in the apatite crystals [348,351], Figure 9l. Fluoridation is a well-known method for preventing dental caries, as it enhances enamel structure and improves its remineralisation; however, a high fluoride concentration can result in enamel fluorosis [692]. Molecular analysis using FTIR and attenuated total reflection (ATR)-FTIR is suitable for studying the interface of different materials, as well as to evaluate their interactions. This approach was utilised to study biomimetic composites, dental materials, dentine and enamel interface, and phosphate and amide groups (corresponding to mineral and organic components respectively [324,325,326,337,347,352,562], Figure 9m), to develop hybrid interfaces. In another study, the distribution of organic and carbonate contents was determined from ancient and modern teeth [341]. This technique benefits from the possibility of analysing both inorganic minerals and organic composition from specific molecular bonds. For example, carious enamel was compared with normal enamel, and a higher intensity of the peaks in the amides and modification of the phosphate group with a shift of the peak were found [171,434]. The technique with a synchrotron was also used to study bovine samples irradiated with a laser, with the observation of the modification of the phosphate group [693,694], as well as the effect of sodium metabisulphite, a reducing agent, in comparison to hydrogen peroxide for tooth whitening [695]. The sample treated with the reducing agent showed a decrease in the intensity of the phosphate group in comparison to the control sample, suggesting a decrease in mineral content. Oral biofilms were also recently analysed, where the molecular composition was obtained from samples with different cariogenic conditions [159], to provide insights into the dynamic processes occurring in dental biofilm with the analysis of lipid, protein, and phosphate.

Synchrotron infrared spectroscopic ellipsometry was carried out on demineralised and remineralised dentine and enamel [419], and variations in the spectra were observed with a different remineralised solution. Specular reflectance synchrotron micro FTIR was also carried out, to study the remineralisation of bovine enamel, to determine the carbonate substitution [569]. The new layers formed during the remineralisation were analysed, the presence of the phosphate group in the spectra acquired in each new layer was identified, and there was a lower intensity of the peaks carbonate group than in the control sample. In comparison to normal enamel, this suggested higher crystallinity of the apatite. An additional spectroscopic technique was explored to study enamel, dentine, and caries using THz scanning near-field imaging [537]. The caries region was observed with lower attenuation of the radiation in comparison to the surrounding material using a near-field image in contrast to a confocal image.

## 4. Conclusions and Perspectives

For more than 35 years, despite limited access, synchrotron radiation facilities with various powerful characterisation techniques (diffraction, scattering, imaging, and spectroscopy) have provided valuable new insights for dental and caries research at different scales. 

To characterise and understand caries formation and progression, a combination of techniques is required, not only to investigate the tissue structure, but also bacterial biofilms, as well as strategies to limit and perhaps repair tissue damage. Each of these topics involves a great number of strategies currently used in ongoing research. Synchrotron-based analyses have led to major advances in the structural biology and physico-chemical and mechanical properties of dental tissues, the caries process, and other dental fields, aiming to improve quality of life. However, there are still some unresolved aspects. Continuously building on the current research will help to better understand the changes in diseased tissue structure and, in turn, its management. Additionally, this review highlights the importance of the applications and approaches carried out on the research of other materials; the knowledge gathered from those approaches can often be transferred to dental caries research, to bring new research opportunities. This can connect methods and analytical results to other research applications.

The direction of research has extended beyond the traditional static analysis, with the incorporation of in situ techniques to monitor processes, as well as the combination of various techniques (XRD, XRF etc.); for instance, correlative imaging analysis with traditional laboratory analysis and with improvement of resolution for instance to target the scale of crystallites with imaging and scattering. This review summarises many recent studies with details and knowledge from state-of-the-art analysis, which could be implemented in future studies to elucidate the phenomenon of caries and explore avenues such as the 3D structure of the nanocrystallites, the motion of atoms occurring during demineralisation, the in situ process of demineralisation by acid from the bacteria using multimodal imaging, in the time domain, as well as energy domains. On the other hand, these techniques can be applied to design and implement new studies for enamel remineralisation and to develop novel biomimetic materials and strategies to repair enamel and dentine. The research on enamel caries is far from being completed. Due to the continuous development of synchrotron facilities, techniques, and devices, it is envisaged that the future will be bright for the research into mineralised tissues. This comprehensive review, thus, is hoped to be of interest to a wide network of researchers and clinicians in the field of cariology and pharmaceutical industries, as well as industries which could benefit from the knowledge transfer of technologies.

All of the aforementioned developments described in this review led to new advances in research on enamel and other mineralised tissues; however, there is a necessity to correlate the findings of synchrotron-based experiments with other techniques such as polarised transmitted light microscopy [145], FIB-SEM [39], (S)TEM [190], radiography/tomography [145], optical coherence tomography (OCT) [696], polarization sensitive OCT [697], APT [698], Raman spectroscopy [168], X-ray photoelectron spectroscopy [699], AFM [700], mechanical tests [701], proton induced X-ray emission (PIXE) [702], neutron [703], time-of-flight secondary ion mass spectroscopy (ToF-SIMS) [704], and indentation [142], to develop a suitable sample preparation workflow and to tackle the disease with a multi-scale multi-correlative characterisation technique strategy.

## Figures and Tables

**Table 1:** Details of the studies carried out on human teeth. Summary of additional studies carried out on the structure of human teeth, with details of findings.

References	Samples	Studies
[39,57,319]	Human enamel, carious and non-carious	Details of the pathways of demineralisation, striations, and surface zone at a voxel size of 325 nm, with more details revealed than with conventional tomography (Figure 8a)
[121]	Human dentine	Details for the tubule density at a pixel size of 370 nm
[359]	Human dentine	3D rendering of cracks in dentine, with an effective pixel size of 300 nm and the influence of dentine tubules on the trajectory of the cracks (Figure 8a)
[318]	Human teeth	Fillings analysis of the quality of the composite in restorations (presence of pores), pixel size of 650 nm
[313]	Human teeth	Reconstruction of the cementum with the observation of tooth cementum annulations (which can be used to estimate age) with a resolution of 615 nm, which has not yet been observed at this resolution with other techniques
[323]	Human dentine	Silver diamine fluoride applied on dentine, to study the mineral precipitation on the sample, tomography taken at a pixel size of ~1.44 µm considered low compared with a vast number of conventional tomography equipment
[314]	Human dentine	Remineralisation of root dentine exposed to demineralising solutions, followed by a fluoride solution with and without EDTA. Tomography was taken at a pixel size of 1.44 µm, and a layer of mineral at the surface of the sample was detected
[311]	Human tooth, cementum	The cementum was imaged and reconstructed with a resolution of 650 nm, with the identification and analysis of incremental layers
[453]	Human enamel and dentine	Data reconstructed with a pixel size of 370 nm with the observation of the tubular structures and enamel region
[391]	Human teeth	Analysis of reconstructed data with a pixel size of 660 nm and 330 nm with details of the microstructure of the cementum (archaeological tooth) and the incremental lines.

**Table 2:** Details of the 3D imaging carried out at high resolution on dental tissue and bone. Details of teeth analysed with high-resolution tomography and the technique used, as well as a few studies on bone.

References	Samples	Resolution or Voxel Size or Effective Pixel Size	Techniques
[357,365]	Human dentine (Figure 8a)	320 nm	Phase-contrast images
[359]	Human dentine	300 nm	X-ray tomography
[109]	Rhinoceros teeth	280 nm	Holotomography
[275]	Cementum of *Kuehneotherium* and *Morganucodon*	280 nm	Propagation distance tomography
[420]	Human dentine	~175 nm	Phase contrast enhanced tomography
[275]	Cementum of *Morganucodon*	10 to 130 nm	Holotomography
[605]	Tooth from a dog, region close to the root	60 nm	Transmission X-ray microscope
[412]	Dentine of human	51 nm	Tomography with Fresnel zone plate setup
[606]	Enamel and dentine of mus musculus and Sorex minutissimus	25 nm	Holotomography
[607]	Human bone	60 nm	Nano-tomography
[608]	Human bone	60 nm	X-ray phase nano-tomography
[609]	Mouse bone	50 nm	Holographic nano-tomography
[610]	Human bone	50 nm	Phase nano-tomography

## Data Availability

Data collected and interpreted in this study are maintained by the authors and can be made available upon request.

## Supplementary Materials

The following supporting information can be downloaded at: https://www.mdpi.com/article/10.3390/dj11040098/s1, Table S1: Analysis of elements in enamel; Table S2: Details of the technique based on dichroism.
