# Supplementary material for: Synchrotron X-ray Studies of the Structural and Functional Hierarchies in Mineralised Human Dental Enamel: A State-of-the-Art Review
Source: Dent J (Basel). 2023 Apr 7;11(4):98. doi: 10.3390/dj11040098 (PMC10137518; doi:10.3390/dj11040098)
Supplement: Supplementary file 1 [file dentistry-11-00098-s001.zip › dentistry-2173806-supplementary.pdf]

Synchrotron X-ray Studies of the Structural and Functional Hierarchies in Mineralised Human Dental Enamel: A State-Of-The-Art Review

Authors

Cyril Besnard*[1], Ali Marie†[1], Sisini Sasidharan†[1], Robert A. Harper[2], Richard M. Shelton[2], Gabriel Landini[2], Alexander M. Korsunsky[1*]

[1] MBLEM, Department of Engineering Science, University of Oxford, Parks Road, Oxford, Oxfordshire, OX1 3PJ, U.K.

[2] School of Dentistry, University of Birmingham, 5 Mill Pool Way, Edgbaston, Birmingham, West Midlands, B5 7EG, U.K.

† These authors contributed equally to this work.

Email addresses:

cyril.besnard@eng.ox.ac.uk, ali.marie@eng.ox.ac.uk, sisini.sasidharan@eng.ox.ac.uk, R.Harper@bham.ac.uk, R.M.Shelton@bham.ac.uk, G.Landini@bham.ac.uk, alexander.korsunsky@eng.ox.ac.uk

* **Corresponding authors:** Alexander M. Korsunsky**,** alexander.korsunsky@eng.ox.ac.uk

Cyril Besnard, cyril.besnard@eng.ox.ac.uk

Supplementary Information (SI)

Table of Contents

Table S1 pp. 3-5

Table S2 pp. 6-9

Supplementary Table S1. Analysis of elements in enamel. Elements that are analysed in human enamel and *A. Africanus* with the techniques used.

Atomic number	Element	References	Technique
1	H	1-10	X-ray diffraction (XRD), neutron, atom probe tomography (APT), Fourier-transform infrared spectroscopy (FTIR), Raman spectroscopy, Raman spectroscopy, element analysis (EA)-thermogravimetric and differential thermal analysis (TGA/DTA), FTIR, time-of-light secondary ion mass spectrometry (ToF-SIMS), FTIR, Raman spectroscopy, Raman spectroscopy
3	Li	11,12	Spark source mass spectrometry (SSMS), laser-ablation inductively coupled-plasma mass spectrometry (LA-ICP-MS)
4	Be	11	Spark plasma mass spectroscopy (SSMS)
5	B	11	SSMS
6	C	1-10,13-28	APT, FTIR, Raman spectroscopy, Raman spectroscopy, EA, X-ray photoelectron spectroscopy- energy dispersive X-ray spectroscopy (XPS-EDS)-FTIR, ToF-SIMS- X-ray micro analyses (XRMA), FITR, Raman spectroscopy, Raman spectroscopy, APT- electron energy loss spectroscopy (EELS), elemental analysis, Raman spectroscopy, Raman spectroscopy, Raman spectroscopy, Raman spectroscopy, XPS, XPS, Raman spectroscopy, EDS, electron energy-loss near-edge structures (ELNES), XRMA, XRMA, SIMS, Raman spectroscopy, IR
7	N	1,2,5-9,14,19,20,23	APT, FTIR, EA, XPS-EDS, ToF-SIMS, FTIR, Raman spectroscopy, elemental analysis, XPS, XPS, ELNES
8	O	1-10,13,15-25,27-30	XRD, neutron, APT, FTIR, Raman spectroscopy, Raman spectroscopy-electron microprobe analysis (EMP) wavelength-dispersive X-ray spectroscopy (WDS), TGA/DTA, XPS-EDS-FTIR, ToF-SIMS, FTIR, Raman spectroscopy, Raman spectroscopy, EELS, Raman spectroscopy, Raman spectroscopy, Raman spectroscopy, Raman spectroscopy, XPS, XPS, Raman spectroscopy, EDS, ELNES, XRMA, XRMA, Raman spectroscopy, IR, EDS, Raman spectroscopy
9	F	1,4,7,11,13,19,20,24,26,29,31,32	APT, EMP (WDS) , ToF-SIMS, SSMS, APT, XPS, XPS, SIMS, SIMS, EDS, secondary ion microanalyzer, magic angle spinning nuclear magnetic resonance (MAS-NMR)
11	Na	1,5,7,13,14,19,20,24,25,28,29,33-36	APT, instrumental neutron activation analysis (INAA), ToF-SIMS-XRMA, APT, wavelength dispersive X-ray fluorescence, XPS, XPS-EDS, X-ray energy dispersive spectroscopy (XEDS), SIMS, XRMA, EDS, EDS, EDS, proton induced X-ray emission (PIXE), LA-ICP-MS, LA-ICP-MS
12	Mg	1,4,5,7,13,14,19,20,25,26,28,33-37	APT, EMP (WDS), inductively-coupled plasma spectrometer (ICP/AES), ToF-SIMS-XRMA, APT-EELS- X-ray absorption near edge structure (XANES), wavelength dispersive X-ray fluorescence, XPS, XPS-EDS, XEDS, SIMS, XRMA, SIMS, EDS, EDS, proton induced X-ray emission (PIXE), LA-ICP-MS, LA-ICP-MS, LA–ICP–MS

13	Al	5,11,34-36	Instrumental neutron activation analysis, SSMS, proton induced X-ray emission (PIXE), LA-ICP-MS, LA-ICP-MS
14	Si	14,19,20	Wavelength dispersive X-ray fluorescence, XPS, XPS-EDS
15	P	1-10,13-25,27-30,33-35,38,39	XRD, neutron, APT, FTIR, Raman spectroscopy, Raman spectroscopy-EMP (WDS), TGA/DTA-ICP/AES, XPS-EDS-FTIR, ToF-SIMS-XRMA, FTIR, Raman spectroscopy, Raman spectroscopy, EELS, Raman spectroscopy, wavelength dispersive X-ray fluorescence, Raman spectroscopy, Raman spectroscopy, Raman spectroscopy, XPS, XPS-EDS, Raman spectroscopy-ICP-EDS, EDS, ELNES-XEDS, XRMA, XRMA, EDS, IR, Raman spectroscopy, Raman spectroscopy, EDS, proton induced X-ray emission (PIXE), LA-ICP-MS, XRF, ICP-MS
16	S	4,11,14,19,34,38	EMP (WDS), SSMS, wavelength dispersive X-ray fluorescence, XPS, proton induced X-ray emission (PIXE), XRF
17	Cl	1,4,5,7,14,19,20,24,25,28,33-35,38	APT, EMP (WDS), instrumental neutron activation analysis, ToF-SIMS-XRMA, wavelength dispersive X-ray fluorescence, XPS, XPS, SIMS, XRMA, EDS, EDS, proton induced X-ray emission (PIXE), LA-ICP-MS, XRF
19	K	4,7,11,14,24,25,28,29,38	EMP (WDS), ToF-SIMS-XRMA, SSMS, wavelength dispersive X-ray fluorescence, SIMS, XRMA, EDS, EDS, XRF
20	Ca	1,4-7,9,12,13,20-25,28,29,33,34,37-47	XRD, neutron, APT, EMP (WDS), instrumental neutron activation analysis, EELS, ToF-SIMS-XRMA, Raman spectroscopy-XANES, LA-ICP-MS, XPS-EDS, XPS-EDS, ICP-EDS, EDS, ELNES-XEDS, XRMA, XRMA, EDS- calcium-ion-specific electrode, EDS, EDS, proton induced X-ray emission (PIXE), LA–ICP–MS, XRF, XRF, XRF, ICP-MS, XANES, XANES (PIC), XRF, particle induced X-ray emission, particle induced x-ray emission, XRF
21	Sc	35,36	LA-ICP-MS, LA-ICP-MS
22	Ti	11,35,36	SSMS, LA-ICP-MS, LA-ICP-MS
23	V	11,34,36,46	SSMS, proton induced X-ray emission (PIXE), LA-ICP-MS, XRF
24	Cr	11,34,36	SSMS, proton induced X-ray emission (PIXE), LA-ICP-MS
25	Mn	11,34,36,45,46,48,49	SSMS, proton induced X-ray emission (PIXE), LA-ICP-MS, XRF, XRF, XRF, XRF
26	Fe	11,14,34,36-38,40,45,46,48,49	SSMS, wavelength dispersive X-ray fluorescence, proton induced X-ray emission (PIXE), LA-ICP-MS, LA–ICP–MS, XRF, XRF, XRF, XRF, XRF, XRF,
27	Co	5,11,36	Instrumental neutron activation analysis, SSMS, LA-ICP-MS
28	Ni	11,34,36	SSMS, proton induced X-ray emission (PIXE), LA-ICP-MS
29	Cu	11,14,34,36-38,40,45,46,48,49	SSMS, wavelength dispersive X-ray fluorescence, proton induced X-ray emission (PIXE), LA-ICP-MS, LA–ICP–MS, XRF, XRF, XRF, XRF, XRF, XRF
30	Zn	5,11,14,19,34-38,40,41,44-46,48,49	Instrumental neutron activation analysis, SSMS, wavelength dispersive X-ray fluorescence, XPS, proton induced X-ray emission (PIXE), LA-ICP-MS, LA-ICP-MS, LA–ICP–MS, XRF, XRF, XRF, XRF, XRF, XRF, XRF
34	Se	11	SSMS

35	Br	11,40,46,48,49	SSMS, XRF, XRF, XRF, XRF
37	Rb	11,36	SSMS, LA-ICP-MS
38	Sr	7,11,12,14,34-37,39-41,44-46,48,49	ToF-SIMS, SSMS, LA-ICP-MS, wavelength dispersive X-ray fluorescence, SIMS, proton induced X-ray emission (PIXE), LA-ICP-MS, LA-ICP-MS, LA–ICP–MS, ICP-MS, XRF, XRF, XRF, XRF, XRF, XRF, XRF
39	Y	36	LA-ICP-MS
40	Zr	11	SSMS
41	Nb	11	SSMS
42	Mo	11	SSMS
47	Ag	11	SSMS
48	Cd	11	SSMS
50	Sn	11	SSMS
51	Sb	11	SSMS
53	I	11,35	SSMS, LA-ICP-MS
56	Ba	11,12,26,35,36,39,45	SSMS, LA-ICP-MS, SIMS, LA-ICP-MS, LA-ICP-MS, ICP-MS, XRF
57	La	36	LA-ICP-MS
71	Lu	36	LA-ICP-MS
73	Ta	5	Instrumental neutron activation analysis
74	W	5	Instrumental neutron activation analysis
80	Hg	35	LA-ICP-MS
82	Pb	11,26,34,36,37,39,40,45,46,48,49	SSMS, SIMS, proton induced X-ray emission (PIXE), LA-ICP-MS, LA–ICP–MS, ICP-MS, XRF, XRF, XRF, XRF, XRF
90	Th	36	LA-ICP-MS
92	U	12,36	LA-ICP-MS, LA-ICP-MS

Supplementary Table S2. Details of the technique based on dichroism. Details of polarization-dependent imaging contrast (PIC) mapping – Photoemission electron microscopy (PEEM) – Dichroism – X-ray polarization – X-ray linear dichroic on mineral, tooth, bone and apatite.

References	Sample	Acquisition location – Analysis – Information	Element and edge	Energy	Map acquired	Resolution Pixel size
50		Theory				
51	Nacre	Synchrotron (SR) – PhotoEmission Electron spectro-Microscopy (PEEM) SPHINX X-PEEM – Synchrotron Radiation Center (SRC)	C K-edge π* and σ*, O π* pre-edge	290.3 and 302 eV, 534 and 531.7 eV	Distribution map	-
52	Red abalone	SR – SPHINX and PEEM-3 – SRC and ALS	O and C π* and σ*, Ca L-edge	534 and 518 eV, 534 and 540 eV, 290.3 and 302 eV, 351.6 and 345 eV	PIC map	-
53	Red abalone	SR – SPHINX X-PEEM – SRC – ALS	C K-edge and O K-edge, Ca L-edge	290.3 and 302 eV, 534 and 531.7 eV, 351.4 and 344.4 eV, 352.7 and 343.7 eV	PIC map	-
54	Sea urchin tooth	SR – PEEM-3 – ALS	Ca L-edge, Ca L_2, Mg K-edge, π* and σ*	352.6 and 342 eV, 1315 and 1309 eV, 290.3 and 302 eV	PIC map π* and σ*	20 nm for Ca
55	Sea urchin tooth	SR – SPHINX X-PEEM – SRC	C K-edge π * and π * and O	290.3 and 302 eV, 534 and 540 eV	X-PEEM map, PIC map	20 nm, resolution
56	Calcite ($CaCO_3$)	SR – PEEM-3 – ALS	C K-edge π* (290.3 eV)	290.3 eV, 280 to 320 eV	X-PEEM images	10 and 30 nm pixel

57		Review				
58	Shells, nacre, $CaCO_3$	SR – PEEM-3 – ALS	C K-edge π* (290.3 eV)	290.3 eV	PIC map	20 nm pixel
59	Shells, nacre	SR – PEEM-3 – ALS	C K-edge π* (290.3 eV)	290.3 eV	PIC map	20 nm pixel
60	Shells	SR – PEEM-3 – ALS	-	-	PIC map	~ 20 nm pixel
61	Calcium carbonate, calcite, vaterite, $CaCO_3$, monohydrocalcite	SR – PEEM-3 – ALS	O K-edge, C K-edge	525 to 555 eV, 534 eV and 290.3 eV	PIC map	20 nm pixel
62	Aragonite, calcite, shell	SR – X-PEEM microscope – Canadian Light Source	C π*, O π*, Ca L-edge	290.3 and 301. eV, 351.3 and 343 eV, 351 and 343 eV, 338 to 370 eV	PIC map	-
63	*Haliotis rufescens*, red abalone, coral, nacre, $CaCO_3$	SR – PEEM-3 – ALS	O K-edge π* (534 eV), Ca L-edge	534 eV, 340 to 360 eV	PIC map for O	20 nm pixel size
64	Tunicate Herdmania momus, Vaterite, $CaCO_3$	SR – PEEM-3 – ALS	O K-edge π* (534 eV)	534 eV	PIC map	20 nm resolution
65	Human bone	SR – X-ray microscope ID21 – ESRF	Ca K-edge	4.02 to 4.15 keV, 4054.0 and 4055.0 eV	XANES in transmission and reflection - XRF	0.8×1.0 μm^2 – 0.7×0.7 μm^2 or of 0.3×0.3 µm2
66	Human tooth, HAp	SR – X-ray microscope ID21 – ESRF	Ca K-edge	4055 and 4053 eV, 4150 eV	Micro XANES	0.8×1.0 μm^2
67	Calcium carbonate, $CaCO_3$	Calculation	Ca L_{23}-edge			-
68	Fossil nacre	SR – PEEM-3 – ALS	O K-edge (534 eV)	525 and 555 eV, 534 eV	PIC map	-
69	Parrotfish teeth, $Ca_5(PO_4)_3F$	SR – PEEM-3 – ALS	Ca L_{23} -edge, C K-edge, O K-edge	352.6 eV and 351.6 eV, 340-360 eV	PIC map	20 nm resolution

70	Coral skeletons $CaCO_3$ amorphous calcium carbonate	SR – Photoelectron emission spectromicroscopy PEEM-3 – ALS	O K-edge π* (534 eV), Ca L-edge	534 eV, 340 to 360 eV,	PIC map	20 nm pixel
71	Coral skeletons $CaCO_3$, synthetic aragonite (SA) spherulites	SR- PEEM-3 – ALS	O K-edge π* (534 eV)	5354 eV	PIC map	-
72		Review				-
73	FAp, HAp, dentine, enameloid, parrotfish, lamellar bone, mouse incisor	SR – PEEM-3 – ALS	Ca L-edge and Ca K-edge	352.8 and 352.4 eV, ±0.2 eV	PIC map	20, 57 and 60 nm pixel
74		Review				-
42	Human tooth Enamel dentine HAp	SR – scanning X-ray microscopy – ESRF	Ca K-edge (4.1 keV)	4.0553 and 4.0533 keV and 4.2 keV, 4.032 to 4.122 keV	Micro XRF maps - XANES	Pixel size 1 or 2 μm – Beam~0.6 × 0.8 μm^2
43	Human tooth enamel	SR – PEEM-3 microscopy beamline – ALS	Ca L-edge (352.6 eV)	352.6 ±0.2 eV	PIC map	22 nm × 22 nm × 3 nm, 57 × 57 × 3 nm
75	Ant head $MgCO_3$	SR – X-ray PhotoEmission Electron spectro-Microscopy (X-PEEM) – ALS	C K-edge, O K-edge π* (534 eV)	280-320 eV 525-555 eV, 534 eV	PIC map for O	-
76	Coral skeletons, spherulitic crystal fibers, $CaCO_3$	SR – PEEM-3 – ALS	O K-edge π* (534 eV)	534 eV	PIC map	20 and 60 nm pixel
77	Sheep enamel HAp, Synthetic fluorapatite dumbbells, and review previous works	SR – PEEM3 microscopy – ALS	Ca L-edge	±0.2 eV	PIC map	60 nm pixel
78	Rat bone	SR – UE56/2 PGM – BESSY II synchrotron light source	Ca 2p edge, Ca $L_{2,3}$ and O K-edge 1s	350 and 535 eV	NEXAFS	Focal spot 1 × 1 mm^2

79	Coral	SR – PEEM – ALS	Ca L-edge and K-edge π*	534 eV	PIC map and PEEM images	Resolution 60 nm
80	Coral skeletons, $CaCO_3$	SR – PEEM-3 – ALS	O K-edge π*	534.5 eV, 1.5 eV below π*, 536.5 eV, 0.5 eV after π*, 536 eV	PIC map and ptychography	Resolution 35 nm and 60 nm
77	Teeth enamel, human, mouse, sheep, parrotfish	SR – PEEM-3 – ALS	Ca L-edge	±0.2 eV	PIC map	-
81	Dolomitic ooid	SR – PEEM – ALS	Carbonate O K-edge π* (534 eV)	534 eV	PIC map	56 nm
82	Review					
83	Biominerals	SR – PEEM-3 – ALS	O K-edge Carbonate π* (534 eV)	534 eV	PIC map	down to 10 nm
84	Sea urchin spine	SR – PEEM – ALS	Ca L-edge	340 to 360 eV	PEEM	~60 nm
85	Dolomitic ooid	SR – PEEM – ALS	Carbonate O K-edge π* (534 eV)	534 eV	PIC map	56 nm
86	Black drum fish teeth	SR – PEEM3 – ALS	Ca L-edge	±0.2 eV	PIC map	-
87	Mice incisor enamel	SR – PEEM3 – ALS	Ca L-edge	±0.2 eV	PIC map	20 and 60 nm
88	Coral skeleton	SR – PEEM – ALS	Ca L-edge	340 and 360 eV	PEEM	24, 54 and 56 nm
89	$CaCO_3$ biominerals	SR – PEEM – ALS	Carbonate O K-edge π* (534 eV	534 eV	PIC map	Resolution 20 nm

References

1 Yun, F. *et al.* Nanoscale pathways for human tooth decay – central planar defect, organic-rich precipitate and high-angle grain boundary. *Biomaterials* **235**, 119748, doi:https://doi.org/10.1016/j.biomaterials.2019.119748 (2020).

2 Ortiz-Ruiz, A. J. *et al.* Structural differences in enamel and dentin in human, bovine, porcine, and ovine teeth. *Annals of Anatomy - Anatomischer Anzeiger* **218**, 7-17, doi:https://doi.org/10.1016/j.aanat.2017.12.012 (2018).

3 Desoutter, A. *et al.* Cross striation in human permanent and deciduous enamel measured with confocal Raman microscopy. *Journal of Raman Spectroscopy* **50**, 548-556, doi:10.1002/jrs.5555 (2019).

4 Li, Z., Al-Jawad, M., Siddiqui, S. & Pasteris, J. D. A mineralogical study in contrasts: highly mineralized whale rostrum and human enamel. *Scientific Reports* **5**, 16511, doi:10.1038/srep16511 (2015).

5 Zenóbio, M. A. F., Tavares, M. S. N., Zenóbio, E. G. & Silva, T. A. Elemental composition of dental biologic tissues: study by means of different analytical techniques. *Journal of Radioanalytical and Nuclear Chemistry* **289**, 161-166, doi:10.1007/s10967-011-1067-1 (2011).

6 Zamudio-Ortega, C. M. *et al.* Morphological, chemical and structural characterisation of deciduous enamel: SEM, EDS, XRD, FTIR and XPS analysis. *European Journal Paediatric Dentistry* **15**, 275-280 (2014).

7 Melin, L. *et al.* XRMA and ToF-SIMS analysis of normal and hypomineralized enamel. *Microscopy and Microanalysis* **21**, 407-421, doi:10.1017/S1431927615000033 (2015).

8 Seredin, P. V. & Melkumov, V. N. Research hydroxyapatite crystals and organic components of hard tooth tissues affected by dental caries using Ftir-microspectroscopy and XRD-microdiffraction. *World Applied Sciences Journal* **31**, 2101-2107 (2014).

9 Goloshchapov, D. *et al.* Raman and XANES spectroscopic study of the influence of coordination atomic and molecular environments in biomimetic composite materials integrated with dental tissue. *Nanomaterials (Basel, Switzerland)* **11**, 3099, doi:10.3390/nano11113099 (2021).

10 Spizzirri, P. G., Cochrane, N. J., Prawer, S. & Reynolds, E. C. A comparative study of carbonate determination in human teeth using Raman spectroscopy. *Caries Research* **46**, 353-360, doi:10.1159/000337398 (2012).

11 Curzon, M. E. J. & Crocker, D. C. Relationships of trace elements in human tooth enamel to dental caries. *Archives of Oral Biology* **23**, 647-653, doi:https://doi.org/10.1016/0003-9969(78)90189-9 (1978).

12 Joannes-Boyau, R. *et al.* Elemental signatures of Australopithecus africanus teeth reveal seasonal dietary stress. *Nature* **572**, 112-115, doi:10.1038/s41586-019-1370-5 (2019).

13 DeRocher, K. A. *et al.* Chemical gradients in human enamel crystallites. *Nature* **583**, 66-71, doi:https://doi.org/10.1038/s41586-020-2433-3 (2020).

14 Teruel, J. d. D., Alcolea, A., Hernández, A. & Ruiz, A. J. O. Comparison of chemical composition of enamel and dentine in human, bovine, porcine and ovine teeth. *Archives of Oral Biology* **60**, 768-775, doi:10.1016/j.archoralbio.2015.01.014 (2015).

15 Buchwald, T. & Buchwald, Z. Assessment of the Raman spectroscopy effectiveness in determining the early changes in human enamel caused by artificial caries. *Analyst* **144**, 1409-1419, doi:10.1039/C8AN01494A (2019).

16 Buchwald, T., Okulus, Z. & Szybowicz, M. Raman spectroscopy as a tool of early dental caries detection–new insights. *Journal of Raman Spectroscopy* **48**, 1094-1102, doi:10.1002/jrs.5175 (2017).

17 Slimani, A. *et al.* Confocal Raman mapping of collagen cross-link and crystallinity of human dentin–enamel junction. *Journal of Biomedical Optics* **22**, 086003 (2017).

18 Al-Obaidi, R. *et al.* Chemical & nano-mechanical study of artificial human enamel subsurface lesions. *Scientific Reports* **8**, 4047, doi:10.1038/s41598-018-22459-7 (2018).

19 Lou, L., Nelson, A. E., Heo, G. & Major, P. W. Surface chemical composition of human maxillary first premolar as assessed by X-ray photoelectron spectroscopy (XPS). *Applied Surface Science* **254**, 6706-6709, doi:https://doi.org/10.1016/j.apsusc.2008.04.085 (2008).

20 Kis, V. K. *et al.* Magnesium incorporation into primary dental enamel and its effect on mechanical properties. *Acta Biomaterialia* **120**, 104-115, doi:https://doi.org/10.1016/j.actbio.2020.08.035 (2021).

21 Abdallah, M.-N. *et al.* Diagenesis-inspired reaction of magnesium ions with surface enamel mineral modifies properties of human teeth. *Acta Biomaterialia* **37**, 174-183, doi:10.1016/j.actbio.2016.04.005 (2016).

22 Zamudio-Ortega, C. M. *et al.* Morphological and chemical changes of deciduous enamel produced by Er:YAG laser, fluoride, and combined treatment. *Photomedicine and Laser Surgery* **32**, 252-259, doi:10.1089/pho.2013.3622 (2014).

23 Srot, V., Bussmann, B., Salzberger, U., Koch, C. T. & van Aken, P. A. Linking microstructure and nanochemistry in human dental tissues. *Microscopy and Microanalysis* **18**, 509-523, doi:10.1017/S1431927612000116 (2012).

24 Sabel, N. *et al.* Elemental composition of normal primary tooth enamel analyzed with XRMA and SIMS. *Swedish Dental Journal* **33**, 75-83 (2009).

25 Sabel, N., Karlsson, A. & Sjölin, L. XRMA analysis and X-ray diffraction analysis of dental enamel from human permanent teeth exposed to hydrogen peroxide of varying pH. *Journal of clinical and experimental dentistry* **11**, e512-e520, doi:10.4317/jced.55618 (2019).

26 Adriaens, A. The role of SIMS in understanding ancient materials. in *Radiation in Art and Archeometry* (eds D. C. Creagh & D. A. Bradley) 180-201 (Elsevier Science B.V., 2000), doi:https://doi.org/10.1016/B978-044450487-6/50055-9.

27 Xu, C., Reed, R., Gorski, J. P., Wang, Y. & Walker, M. P. The distribution of carbonate in enamel and its correlation with structure and mechanical properties. *Journal of Materials Science* **47**, 8035-8043, doi:10.1007/s10853-012-6693-7 (2012).

28 LeGeros, R. Z., Sakae, T., Bautista, C., Retino, M. & LeGeros, J. P. Magnesium and carbonate in enamel and synthetic apatites. *Advances in Dental Research* **10**, 225-231, doi:10.1177/08959374960100021801 (1996).

29 Cakir, F. Y., Korkmaz, Y., Firat, E., Oztas, S. S. & Gurgan, S. Chemical analysis of enamel and dentin following the application of three different at-home bleaching systems. *Operative Dentistry* **36**, 529-536, doi:10.2341/11-050-l (2011).

30 Buchwald, T. & Okulus, Z. Determination of storage solutions influence on human enamel by Raman spectroscopy. *Vibrational Spectroscopy* **96**, 118-124, doi:https://doi.org/10.1016/j.vibspec.2018.04.003 (2018).

31 Petersson, L. G., Odelius, H., Lodding, A., Larsson, S. J. & Frostell, G. Ion probe study of fluorine gradients in outermost layers of human enamel. *Journal of Dental Research* **55**, 980-990, doi:10.1177/00220345760550065001 (1976).

32 Mohammed, N. R. *et al.* Effects of fluoride on in vitro enamel demineralization analyzed by ^{19}F MAS-NMR. *Caries Research* **47**, 421-428, doi:10.1159/000350171 (2013).

33 Gómez, B. F. *et al.* Effect of flavored water on the morphological and chemical composition of human dental enamel microestructure in vitro *South Florida Journal of Development* **2**, 1724-1732, doi:https://doi.org/10.46932/sfjdv2n2-047 (2021).

34 Anwar Chaudhri, M. & Ainsworth, T. Applications of PIXE to studies in dental and mental healths. *Nuclear Instruments and Methods* **181**, 333-336, doi:https://doi.org/10.1016/0029-554X(81)90632-7 (1981).

35 Cucina, A., Dudgeon, J. & Neff, H. Methodological strategy for the analysis of human dental enamel by LA-ICP-MS. *Journal of Archaeological Science* **34**, 1884-1888, doi:https://doi.org/10.1016/j.jas.2007.01.004 (2007).

36 Guede, I. *et al.* Analyses of human dentine and tooth enamel by laser ablation-inductively coupled plasma-mass spectrometry (LA-ICP-MS) to study the diet of medieval Muslim individuals from Tauste (Spain). *Microchemical Journal* **130**, 287-294, doi:https://doi.org/10.1016/j.microc.2016.10.005 (2017).

37 Kang, D., Amarasiriwardena, D. & Goodman, A. H. Application of laser ablation–inductively coupled plasma-mass spectrometry (LA–ICP–MS) to investigate trace metal spatial distributions in human tooth enamel and dentine growth layers and pulp. *Analytical and Bioanalytical Chemistry* **378**, 1608-1615, doi:10.1007/s00216-004-2504-6 (2004).

38 Harris, H. H., Vogt, S., Eastgate, H. & Lay, P. A. A link between copper and dental caries in human teeth identified by X-ray fluorescence elemental mapping. *JBIC Journal of Biological Inorganic Chemistry* **13**, 303-306, doi:10.1007/s00775-007-0321-z (2008).

39 Liu, H.-Y. *et al.* Study of P, Ca, Sr, Ba and Pb levels in enamel and dentine of human third molars for environmental and archaeological research. *Advances in Anthropology* **3**, 71-77, doi:10.4236/aa.2013.32010 (2013).

40 Carvalho, M. L., Casaca, C., Marques, J. P., Pinheiro, T. & Cunha, A. S. Human teeth elemental profiles measured by synchrotron x-ray fluorescence: dietary habits and environmental influence. *X-Ray Spectrometry* **30**, 190-193, doi:10.1002/xrs.487 (2001).

41 Dean, M. C., Spiers, K. M., Garrevoet, J. & Le Cabec, A. Synchrotron X-ray fluorescence mapping of Ca, Sr and Zn at the neonatal line in human deciduous teeth reflects changing perinatal physiology. *Archives of Oral Biology* **104**, 90-102, doi:https://doi.org/10.1016/j.archoralbio.2019.05.024 (2019).

42 Hesse, B., Stier, D., Cotte, M., Forien, J.-B. & Zaslansky, P. Polarization induced contrast X-ray fluorescence at submicrometer resolution reveals nanometer apatite crystal orientations across entire tooth sections. *Biomedical Optics Express* **10**, 18-28, doi:10.1364/BOE.10.000018 (2019).

43 Beniash, E. *et al.* The hidden structure of human enamel. *Nature Communications* **10**, 4383, doi:https://doi.org/10.1038/s41467-019-12185-7 (2019).

44 Anjos, M. J. *et al.* Elemental mapping of teeth using µSRXRF. *Nuclear Instruments and Methods in Physics Research Section B: Beam Interactions with Materials and Atoms* **213**, 569-573, doi:https://doi.org/10.1016/S0168-583X(03)01673-2 (2004).

45 Carvalho, M. L., Marques, A. F., Marques, J. P. & Casaca, C. Evaluation of the diffusion of Mn, Fe, Ba and Pb in Middle Ages human teeth by synchrotron microprobe X-ray fluorescence. *Spectrochimica Acta Part B: Atomic Spectroscopy* **62**, 702-706, doi:https://doi.org/10.1016/j.sab.2007.02.011 (2007).

46 Carvalho, M. L., Marques, J. P., Marques, A. F. & Casaca, C. Synchrotron microprobe determination of the elemental distribution in human teeth of the Neolithic period. *X-Ray Spectrometry* **33**, 55-60, doi:https://doi.org/10.1002/xrs.705 (2004).

47 Besnard, C. *et al.* Nanoscale correlative X-ray spectroscopy and ptychography of carious dental enamel. *Materials & Design* **224**, 111272, doi:https://doi.org/10.1016/j.matdes.2022.111272 (2022).

48 Marques, A. F., Marques, J. P., Casaca, C. & Carvalho, M. L. X-ray microprobe synchroton radiation X-ray fluorescence application on human teeth of renal insufficiency patients. *Spectrochimica Acta Part B: Atomic Spectroscopy* **59**, 1675-1680, doi:https://doi.org/10.1016/j.sab.2004.07.017 (2004).

49 Carvalho, M. L. *et al.* Analysis of human teeth and bones from the chalcolithic period by X-ray spectrometry. *Nuclear Instruments and Methods in Physics Research Section B: Beam Interactions with Materials and Atoms* **168**, 559-565, doi:https://doi.org/10.1016/S0168-583X(00)00049-5 (2000).

50 Brouder, C. Angular dependence of X-ray absorption spectra. *Journal of Physics: Condensed Matter* **2**, 701-738, doi:10.1088/0953-8984/2/3/018 (1990).

51 Metzler, R. A. *et al.* Architecture of columnar nacre, and implications for its formation mechanism. *Physical Review Letters* **98**, 268102 (2007).
52 Gilbert, P. U. P. A. *et al.* Gradual ordering in red abalone nacre. *Journal of the American Chemical Society* **130**, 17519-17527, doi:10.1021/ja8065495 (2008).
53 Metzler, R. A. *et al.* Polarization-dependent imaging contrast in abalone shells. *Physical Review B* **77**, 064110-064112-064110-064119 (2008).
54 Killian, C. E. *et al.* Mechanism of calcite co-orientation in the sea urchin tooth. *Journal of the American Chemical Society* **131**, 18404-18409, doi:10.1021/ja907063z (2009).
55 Ma, Y. *et al.* The grinding tip of the sea urchin tooth exhibits exquisite control over calcite crystal orientation and Mg distribution. *Proceedings of the National Academy of Sciences of the United States of America* **106**, 6048-6053, doi:10.1073/pnas.0810300106 (2009).
56 Gilbert, P. U. P. A., Young, A. & Coppersmith, S. N. Measurement of *c*-axis angular orientation in calcite ($CaCO_3$) nanocrystals using X-ray absorption spectroscopy. *Proceedings of the National Academy of Sciences* **108**, 11350-11355, doi:10.1073/pnas.1107917108 (2011).
57 Gilbert, P. U. P. A. Polarization-dependent imaging contrast (PIC) mapping reveals nanocrystal orientation patterns in carbonate biominerals. *Journal of Electron Spectroscopy and Related Phenomena* **185**, 395-405, doi:https://doi.org/10.1016/j.elspec.2012.06.001 (2012).
58 Olson, I. C., Kozdon, R., Valley, J. W. & Gilbert, P. U. P. A. Mollusk shell nacre ultrastructure correlates with environmental temperature and pressure. *Journal of the American Chemical Society* **134**, 7351-7358, doi:10.1021/ja210808s (2012).
59 Olson, I. C. *et al.* Crystal nucleation and near-epitaxial growth in nacre. *Journal of Structural Biology* **184**, 454-463, doi:https://doi.org/10.1016/j.jsb.2013.10.002 (2013).
60 Olson, I. C. *et al.* Crystal lattice tilting in prismatic calcite. *Journal of Structural Biology* **183**, 180-190, doi:https://doi.org/10.1016/j.jsb.2013.06.006 (2013).
61 DeVol, R. T. *et al.* Oxygen spectroscopy and polarization-dependent imaging contrast (PIC)-mapping of calcium carbonate minerals and biominerals. *The Journal of Physical Chemistry B* **118**, 8449-8457, doi:10.1021/jp503700g (2014).
62 Metzler, R. A. & Rez, P. Polarization dependence of aragonite calcium L-edge XANES spectrum indicates c and b axes orientation. *The Journal of Physical Chemistry B* **118**, 6758-6766, doi:10.1021/jp503565e (2014).
63 DeVol, R. T. *et al.* Nanoscale transforming mineral phases in fresh nacre. *Journal of the American Chemical Society* **137**, 13325-13333, doi:10.1021/jacs.5b07931 (2015).
64 Pokroy, B. *et al.* Narrowly distributed crystal orientation in biomineral vaterite. *Chemistry of Materials* **27**, 6516-6523, doi:10.1021/acs.chemmater.5b01542 (2015).
65 Hesse, B. *et al.* Full-field calcium K-edge X-ray absorption near-edge structure spectroscopy on cortical bone at the micron-scale: polarization effects reveal mineral orientation. *Analytical Chemistry* **88**, 3826-3835, doi:10.1021/acs.analchem.5b04898 (2016).
66 Hesse, B., Zaslansky, P., Salome, M., Castillo, H. & Cotte, M. Angular-dependent absorption spectroscopy reveals apatite crystal orientation in human teeth. in *XRM2016: 13th International Conference on X-Ray Microscopy.* (Oxford, United Kingdom, 2016).
67 Krüger, P. & Natoli, C. R. Theory of x-ray absorption and linear dichroism at the Ca L_{23}-edge of $CaCO_3$. *Journal of Physics: Conference Series* **712**, 012007, doi:10.1088/1742-6596/712/1/012007 (2016).
68 Gilbert, P. U. P. A. *et al.* Nacre tablet thickness records formation temperature in modern and fossil shells. *Earth and Planetary Science Letters* **460**, 281-292, doi:https://doi.org/10.1016/j.epsl.2016.11.012 (2017).
69 Marcus, M. A. *et al.* Parrotfish teeth: stiff biominerals whose microstructure makes them tough and abrasion-resistant to bite stony corals. *ACS Nano* **11**, 11856-11865, doi:10.1021/acsnano.7b05044 (2017).

70 Mass, T. *et al.* Amorphous calcium carbonate particles form coral skeletons. *Proceedings of the National Academy of Sciences* **114**, E7670-E7678, doi:10.1073/pnas.1707890114 (2017).

71 Sun, C.-Y. *et al.* Spherulitic growth of coral skeletons and synthetic aragonite: nature's three-dimensional printing. *ACS Nano* **11**, 6612-6622, doi:10.1021/acsnano.7b00127 (2017).

72 Gilbert, P. U. P. A. Polarization-dependent imaging contrast (PIC) mapping in 2018. *Microscopy and Microanalysis* **24**, 454-457, doi:10.1017/S1431927618014514 (2018).

73 Stifler, C. A. *et al.* X-ray linear dichroism in apatite. *Journal of the American Chemical Society* **140**, 11698-11704, doi:https://doi.org/10.1021/jacs.8b05547 (2018).

74 Gilbert, P. U. P. A. & Stifler, C. A. See-through teeth, clearly. *Matter* **1**, 27-29, doi:https://doi.org/10.1016/j.matt.2019.06.015 (2019).

75 Li, H. *et al.* Biomineral armor in leaf-cutter ants. *bioRxiv*, 2020.2005.2018.102962, doi:10.1101/2020.05.18.102962 (2020).

76 Sun, C.-Y. *et al.* Crystal nucleation and growth of spherulites demonstrated by coral skeletons and phase-field simulations. *Acta Biomaterialia*, doi:https://doi.org/10.1016/j.actbio.2020.06.027 (2020).

77 Stifler, C. A. *et al.* Crystal misorientation correlates with hardness in tooth enamels☆. *Acta Biomaterialia* **120**, 124-134, doi:https://doi.org/10.1016/j.actbio.2020.07.037 (2021).

78 Konashuk, A. S., Brykalova, X. O., Kornilov, N. N., Filatova, E. O. & Pavlychev, A. A. Hierarchy-induced X-ray linear dichroism in cortical bone. *Emergent Materials* **3**, 515-520, doi:10.1007/s42247-020-00105-1 (2020).

79 Sun, C.-Y. *et al.* From particle attachment to space-filling coral skeletons. *Proceedings of the National Academy of Sciences*, 202012025, doi:10.1073/pnas.2012025117 (2020).

80 Lo, Y. H. *et al.* X-ray linear dichroic ptychography. *Proceedings of the National Academy of Sciences* **118**, e2019068118, doi:10.1073/pnas.2019068118 (2021).

81 Wilcots, J., Gilbert, P. U. & Bergmann, K. D. Nanoscale crystal fabric of primary Ediacaran dolomite. *Earth and Space Science Open Archive*, 15, doi:doi:10.1002/essoar.10507750.2 (2021).

82 Gránásy, L. *et al.* Phase-field modeling of biomineralization in mollusks and corals: microstructure vs formation mechanism. *JACS Au* **1**, 1014-1033, doi:https://doi.org/10.1021/jacsau.1c00026 (2021).

83 Lew, A. J., Stifler, C. A., Schmidt, C. A., Buehler, M. J. & Gilbert, P. U. P. A. Small-misorientation toughness in biominerals evolved convergently. *arXiv.org*, arXiv:2108.07877 (2021).

84 Stifler, C. A., Killian, C. E. & Gilbert, P. U. P. A. Evidence for a liquid precursor to biomineral formation. *Crystal Growth & Design* **21**, 6635-6641, doi:10.1021/acs.cgd.1c00865 (2021).

85 Wilcots, J., Gilbert, P. U. & Bergmann, K. D. Nanoscale crystal fabric preserved in dolomite ooids at the onset of the Shuram Excursion. *Earth and Space Science Open Archive*, 19, doi:doi:10.1002/essoar.10507750.3 (2021).

86 Deng, Z. *et al.* Black drum fish teeth: built for crushing mollusk shells. *Acta Biomaterialia* **137**, 147-161, doi:https://doi.org/10.1016/j.actbio.2021.10.023 (2022).

87 Stifler, C. A., Yamazaki, H., Gilbert, P. U. P. A., Margolis, H. C. & Beniash, E. Loss of biological control of enamel mineralization in amelogenin-phosphorylation-deficient mice. *Journal of Structural Biology* **214**, 107844, doi:https://doi.org/10.1016/j.jsb.2022.107844 (2022).

88 Schmidt, C. A. *et al.* Faster crystallization during coral skeleton formation correlates with resilience to ocean acidification. *Journal of the American Chemical Society* **144**, 1332-1341, doi:10.1021/jacs.1c11434 (2022).

89 Gilbert, P. *et al.* Convergent, slightly misoriented crystals toughen corals and seashells. *Research Square*, doi:https://doi.org/10.21203/rs.3.rs-1879515/v1 (2022).
